# Evaluation of the Leap Motion Controller during the performance of visually-guided upper limb movements

**DOI:** 10.1371/journal.pone.0193639

**Published:** 2018-03-12

**Authors:** Ewa Niechwiej-Szwedo, David Gonzalez, Mina Nouredanesh, James Tung

**Affiliations:** 1 Department of Kinesiology, University of Waterloo, Waterloo, Canada; 2 Department of Mechanical and Mechatronics Engineering, University of Waterloo, Waterloo, Canada; University of Exeter, UNITED KINGDOM

## Abstract

Kinematic analysis of upper limb reaching provides insight into the central nervous system control of movements. Until recently, kinematic examination of motor control has been limited to studies conducted in traditional research laboratories because motion capture equipment used for data collection is not easily portable and expensive. A recently developed markerless system, the Leap Motion Controller (LMC), is a portable and inexpensive tracking device that allows recording of 3D hand and finger position. The main goal of this study was to assess the concurrent reliability and validity of the LMC as compared to the Optotrak, a criterion-standard motion capture system, for measures of temporal accuracy and peak velocity during the performance of upper limb, visually-guided movements. In experiment 1, 14 participants executed aiming movements to visual targets presented on a computer monitor. Bland-Altman analysis was conducted to assess the validity and limits of agreement for measures of temporal accuracy (movement time, duration of deceleration interval), peak velocity, and spatial accuracy (endpoint accuracy). In addition, a one-sample t-test was used to test the hypothesis that the error difference between measures obtained from Optotrak and LMC is zero. In experiment 2, 15 participants performed a Fitts’ type aiming task in order to assess whether the LMC is capable of assessing a well-known speed-accuracy trade-off relationship. Experiment 3 assessed the temporal coordination pattern during the performance of a sequence consisting of a reaching, grasping, and placement task in 15 participants. Results from the t-test showed that the error difference in temporal measures was significantly different from zero. Based on the results from the 3 experiments, the average temporal error in movement time was 40±44 ms, and the error in peak velocity was 0.024±0.103 m/s. The limits of agreement between the LMC and Optotrak for spatial accuracy measures ranged between 2–5 cm. Although the LMC system is a low-cost, highly portable system, which could facilitate collection of kinematic data outside of the traditional laboratory settings, the temporal and spatial errors may limit the use of the device in some settings.

## Introduction

Upper limb reaching movements are an essential component of most daily activities. In fact, humans make hundreds of reaching movements as they reach towards objects and manipulate them throughout the day. Kinematic assessment provides important insight into the neural control of upper limb movements during development, aging, and in case of neuropathologies, such as stroke [1,2] or concussions [3]. To date, kinematic assessments have largely been conducted in laboratory settings using highly specialized and expensive equipment, such as the Optotrak system (NDI, Waterloo, Canada) or the Vicon system (Vicon, Denver, USA) among others. Recently, portable and relatively low-cost devices have become available, which could facilitate assessment of motor control in a wide range of settings that are not constrained to the laboratory. For example, the Leap Motion Controller system (LMC) (Leap Motion Inc., San Francisco, USA) is a commercially available, easily portable, tracking device that can capture 3D upper limb movements. As stated in the company’s website (https://developer.leapmotion.com/sdk/v2), the LMC uses two cameras and 3 infrared light emitting diodes (LEDs–wavelength 850 nm) to track the position of the palm of the hand, wrist orientation, and the five digits. Proprietary software provided by the developers can be used to capture and save the 3D position of each segment with the origin of the coordinate frame centered on the LMC. A custom Java application can be used to save the recorded data for further analysis. Although the sampling rate of the LMC cannot be set by the user, the LMC offers several important advantages: cost (~$100 USD), portability, minimal set-up time, and a software development kit (SDK) to create custom acquisition applications, which make this device potentially useful for assessment of upper limb movements in clinical settings outside the traditional research laboratory. However, it is important to establish the validity of the LMC system using criterion-standard motion tracking devices. Importantly, the comparison has to be performed using tasks and outcome measures that have clinical utility. Therefore, the goal of this study was to evaluate the reliability and validity of the LMC system compared to a research-grade Optotrak motion capture system (NDI, Waterloo) during the performance of upper limb reaching and grasping movements.

Optimal motor performance can be operationally defined as movements that are performed rapidly and accurately, while minimizing the energy and mental costs [4]. While coarse outcome measures, such as movement duration and accuracy, provide an overall index of motor performance, kinematic outcome measures recorded with a motion capture device provide additional insight into motor planning and feedback control processes. Movement kinematics, such as peak velocity, have been used to infer motor planning, while the duration of the deceleration phase has been used to infer feedback processes [5–9]. Studies conducted in research laboratories using state-of-the-art motion capture systems, such as the Optotrak and others, have established the usefulness of such kinematic measures for the assessment of motor control during development [10–12], aging [13], and with neuropathological populations [5]. However, state-of-the-art motion capture systems are limited by high cost, lack of portability (i.e., fixed location), and high technical expertise required to use the equipment.

Since the LMC system has been released a few years ago, several research reports have evaluated its spatial accuracy during the performance of upper limb tasks. One of the first reports focused on testing accuracy and repeatability during static and dynamic tasks using an industrial robot [14]. According to the initial report, the instrument’s accuracy was 0.2 mm during static tests, and <2.5 mm during dynamic tests. These results were corroborated by Guna and colleagues [15] in a study that evaluated the LMC system using a plastic arm model to simulate human arm motion. They showed that LMC’s accuracy is <0.5 mm when testing at locations ±20 cm along the azimuth, up to 30 cm in depth and elevation. In contrast to these promising results using simulated human movement, more recent studies reported significantly greater spatial error for actual pointing movements performed to targets presented on a computer monitor. For example, Bachmann and colleagues [16] used a Fitts tapping task and found significantly greater error rates, defined as the percentage of out-of-target selections, for the LMC (7.2%) compared to a standard mouse device (2.3%). They also found that movement time was significantly longer with the LMC compared with the mouse movements (945 vs. 565 ms). One limitation of this research is that the trials recorded with the mouse or with the LMC were not recorded simultaneously. This limitation was addressed by Tung and colleagues [17] in a study where pointing movements were recorded simultaneously with the LMC and the Optotrak. This study focused on measuring the static spatial accuracy of pointing movements to 15 targets displayed in a 5x3 grid (targets along the azimuth were separated by 12 cm, targets along the elevation were separated by 11 cm). The spatial error was dependent on target location, with greater error (root mean square error [RMS] >20 mm) obtained for movements to the extreme left, right, or bottom of the display. However, RMS error was <15 mm to targets presented within ±12 cm along the azimuth. Bland-Altman analysis demonstrated a mean difference in position accuracy between the Optotrak and LMC systems as 2.39 mm, with the 95% limits of agreement between -22.02 and +26.80 mm. More recently, Smeragliuolo and colleagues examined the efficacy of the LMC system for the assessment of wrist motion [18]. Results showed that the LMC system provides a valid measurement (i.e., within 10% of the full range of motion) for wrist flexion/extension and radial/ulnar deviation (RMS error: 11.6° and 12.4°, respectively).

In summary, previous studies evaluating the efficacy of the LMC system have focused on spatial accuracy, and to our knowledge, there have been no studies examining temporal accuracy or limb velocity. Given the advantages of the LMC system (i.e., cost and portability), the main goal of this study was to assess the reliability and validity of LMC for measuring movement duration and peak velocity in a series of 3 experiments. In experiment 1, the Optotrak and the LMC systems were used simultaneously as participants performed visually-guided reaching movements, which allowed us to evaluate concurrent validity and the limits of agreement between the two systems. A Fitts’ type aiming task was used in experiment 2 to examine the LMC’s capability to assess speed-accuracy trade-off. Finally, experiment 3 assessed a temporal coordination pattern during a sequencing task consisting of reaching, grasping and placement.

## Methods

The methodology common to all 3 experiments is described first, and each experimental protocol is described separately in subsequent sections. The protocols used in this study were approved by the Office of Research Ethics at the University of Waterloo. All participants signed a consent form prior to the start of the experiment.

### Apparatus

The visual stimulus was presented on an LED monitor (ViewSonic, resolution 1920x1080 @85Hz). The fixation stimulus was a white cross (0.25°) presented at eye level and aligned with the midline. The stimulus for visually-guided aiming was a white circle (0.25°), which was presented at 4 locations along the azimuth (±5°, ±10°). Stimulus presentation was controlled by a custom VPixx script (VPixx Technologies, Quebec, Canada), which randomized the order of presentation.

**Fig 1:**
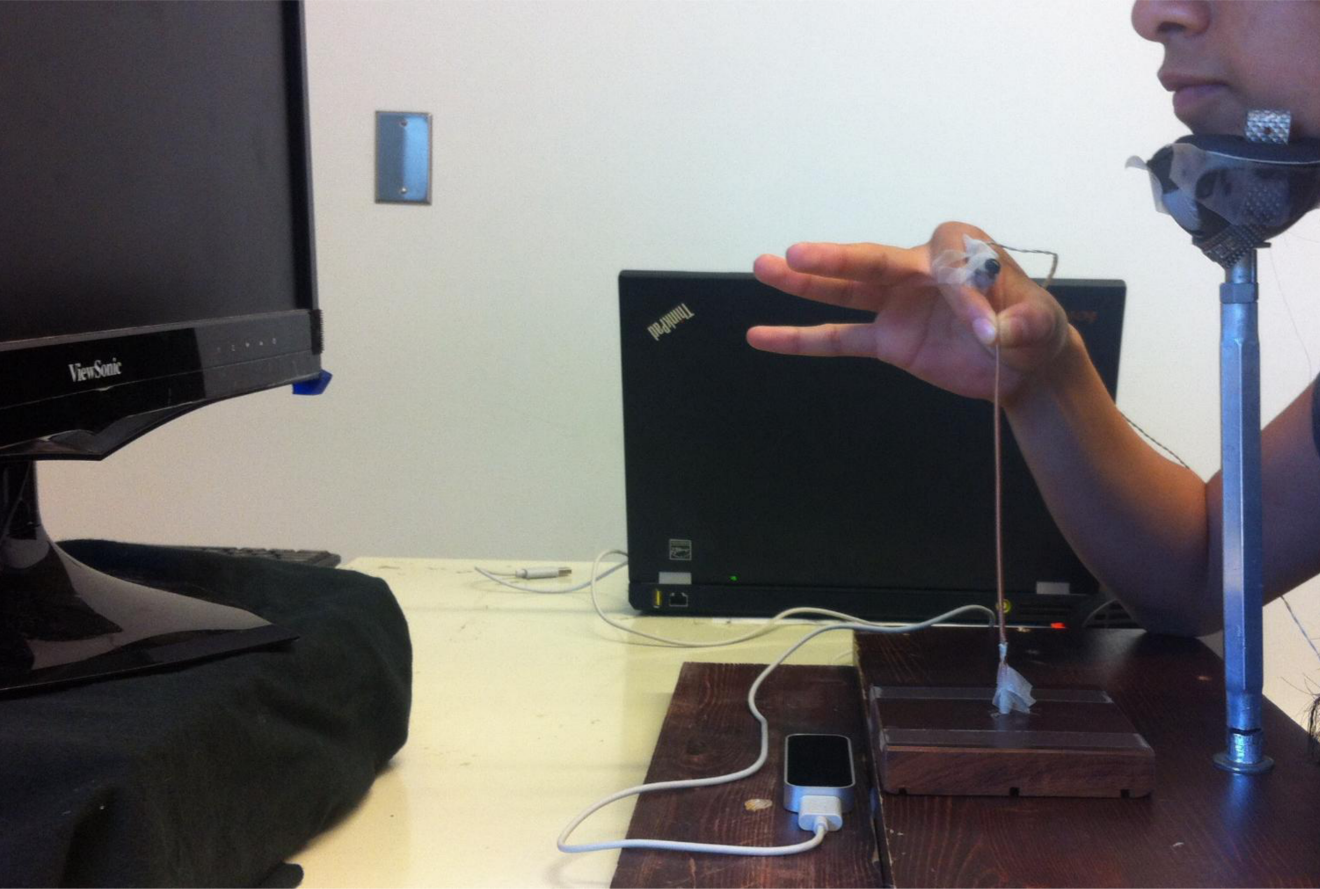
Picture illustrating the experimental set up. The IREDs were placed on the participant’s index finger (intermediate phalanx) and the radial aspect of the wrist. At the initiation of each trial participants were holding the tip of the needle with their index finger and thumb while the other fingers were extended. When movement was initiated, the index finger was extended and the other fingers flexed slightly.

Limb kinematic data were captured with 2 motion capture systems: Optotrak 3D Investigator system (NDI, Waterloo) and the LMC system (software version 2.3.1, Leap Motion Inc, San Francisco, USA). To record the Optotrak data, 2 infrared emitting diodes (IRED) were placed on the participant’s index finger (intermediate phalanx) and the radial aspect of the wrist. Data were collected using the First Principles software (NDI, Waterloo, Canada) at a sampling rate of 50 Hz. The coordinate system was defined as follows: x-axis–azimuth, y-axis–elevation, z-axis–depth). Participants were seated at a table, with their dominant hand placed at body midline, 10 cm above the table. The LMC was placed on the table, aligned with the participant’s midline along the azimuth, directly 10 cm away in front of the start hand position, and 30 cm away from the display monitor in depth (please see Fig 1). This location is consistent with the recommended placement position from the LMC’s user guide (https://developer.leapmotion.com/sdk/v2), and based on our pilot studies, this location was optimal for capturing the maximum amount of data. The coordinate axes were aligned with the Optotrak system. The LMC system does not allow the sampling rate to be specified by the user so the sampling rate was calculated offline. The LMC kinematic data were collected and recorded for offline processing using a custom Java application using the LMC Motion SDK (Core Asset 4.1.1) using a ThinkPad T430, Lenovo laptop (Intel Core i5-3230M Processor– 3.20GHz, 3MB Cache, 1600 MHz).

### Experiment #1: Visually-guided reaching

#### Participants

Fourteen young adults (23.5± 5yrs; 7 males) recruited from the University of Waterloo participated in the first experiment. All participants had normal visual acuity (20/20), and reported to be right-hand dominant. None of the participants reported any neuromuscular symptoms, or previous injuries to the upper limb.

#### Procedure

Participants were seated 45 cm in front of the display computer with their chin in a chin-rest, which ensured a standard viewing distance and a comfortable reaching movement for all participants. Participants were instructed to wear a short-sleeved shirt to ensure that loose clothing will not interfere with data acquisition. As shown in Fig 1, the start position for the hand was at midline along azimuth, 30 cm from the display, and raised 10 cm above the table. The position was standardized by asking participants to hold a thin, blunt needle with their index finger and thumb. Participants were asked to extend the other 3 fingers in order to facilitate maximum data capture by the LMC system (Fig 1). Participants were instructed to fixate the fixation cross for 1–1.5 sec, and to make a fast and accurate reaching movement to touch a visual stimulus, which appeared after the fixation disappeared, and which was presented randomly at ±5°, ±10° from the fixation along the azimuth. Data were acquired using 2 different computers as described in the Apparatus section, and the collection was synchronized by the experimenter pressing 2 buttons on the respective computers to initiate collection with the Optotrak and the LMC systems. Data collection was initiated prior to the onset of the fixation cross when the experimenter ensured that both, the Optotrak and the LMC were tracking the fingers.

Participants performed 20 trials for each target location for a total of 80 trials. At the end of the collection protocol, a calibration trial was collected where participants were asked to point to each of the 4 visual targets as accurately as possible, without time constraints. Accuracy was enforced by the experimenter who ensured that the participant’s finger was placed on the visual target and held at the location for 2–3 s. The collection procedure lasted approximately 30 min.

#### Data processing

Data were analysed offline using a custom Matlab script (Mathworks Inc, Natick, MA, USA). The average sampling rate across the 14 participants for the LMC system was 89.45±28.03 Hz (range 51–113 Hz; Table 1). Due to the variable sampling rate, LMC position data were first fitted using a cubic spline function and resampled at 50 Hz (using matlab function pchip). Trials with more than 40 ms (i.e. 2 frames) of missing data were excluded from further analysis.

**Table 1:** Average sampling rate obtained with the LMC, and the number of usable trials (≤ 40 ms of missing data) obtained from the LMC and Optotrak.

Participant	Average sampling rate (Hz) obtained from LMC	# of usable LMC trials	# of usable Optotrak trials
1	57.14	66/80 (83%)	79/80 (99%)
2	113.51	35/80 (44%)	76/80 (95%)
3	112.11	67/80 (84%)	78/80 (98%)
4	51.76	49/80 (61%)	78/80 (98%)
5	108.81	64/80 (80%)	66/80 (83%)
6	113.51	64/80 (80%)	77/80 (96%)
7	113.51	61/80 (76%)	79/80 (99%)
8	113.38	68/80 (85%)	79/80 (99%)
9	113.51	71/80 (89%)	75/80 (94%)
10	56.49	52/80 (65%)	70/80 (88%)
11	113.38	62/80 (77%)	79/80 (99%)
12	65.06	55/80 (69%)	70/80 (88%)
13	60.10	30/80 (38%)	77/80 (96%)
14	60.10	58/80 (73%)	77/80 (96%)
**MEAN**	**89.45 ± 28.03**	**57/80 (71%)**	**76/80 (95%)**

**Fig 2:**
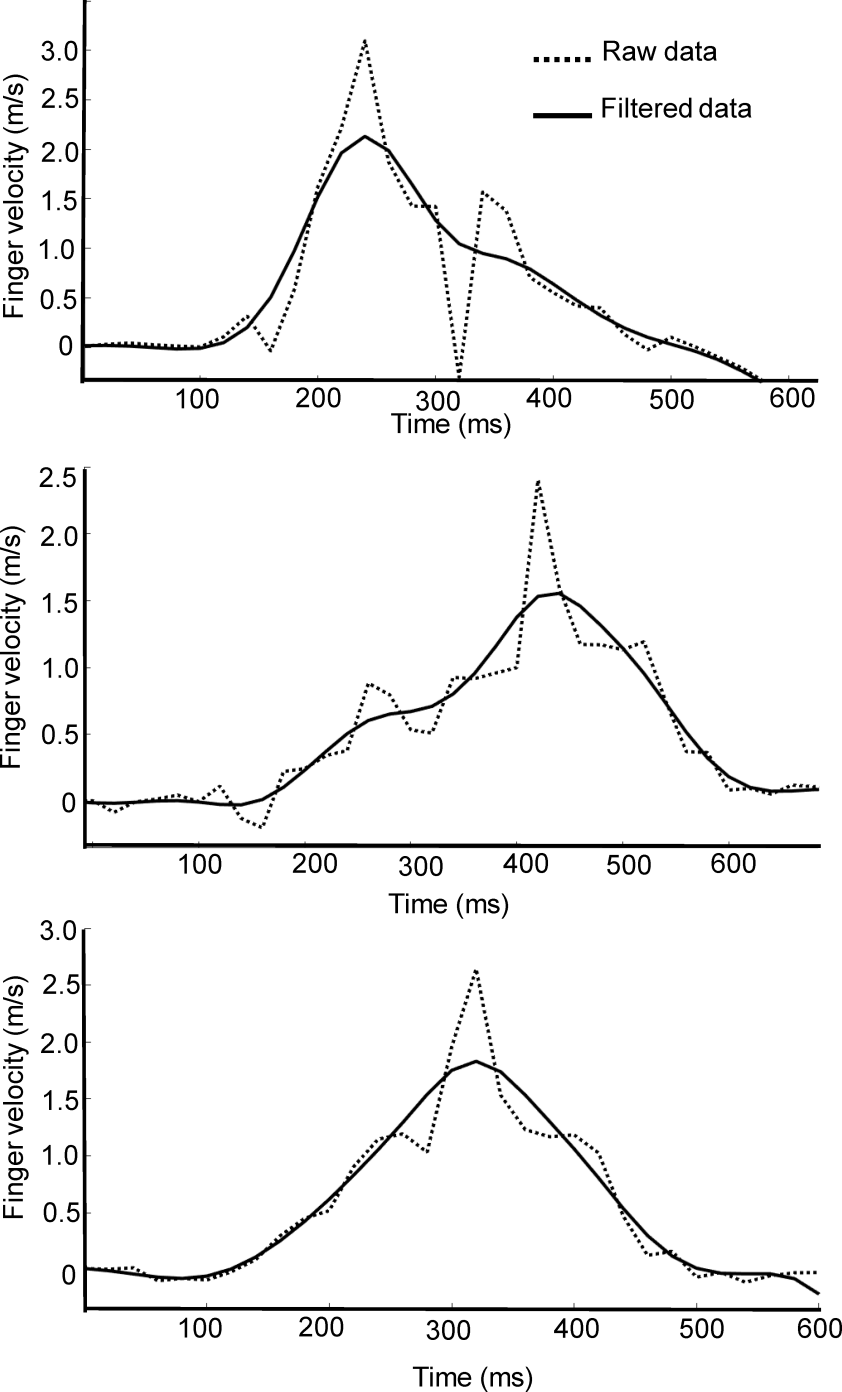
Three trials showing reach velocity data obtained along the primary direction of movement (i.e., depth axis) from the LMC. High frequency component was evident in the raw velocity plots for some trials, which were excluded from subsequent analysis.

**Fig 3:**
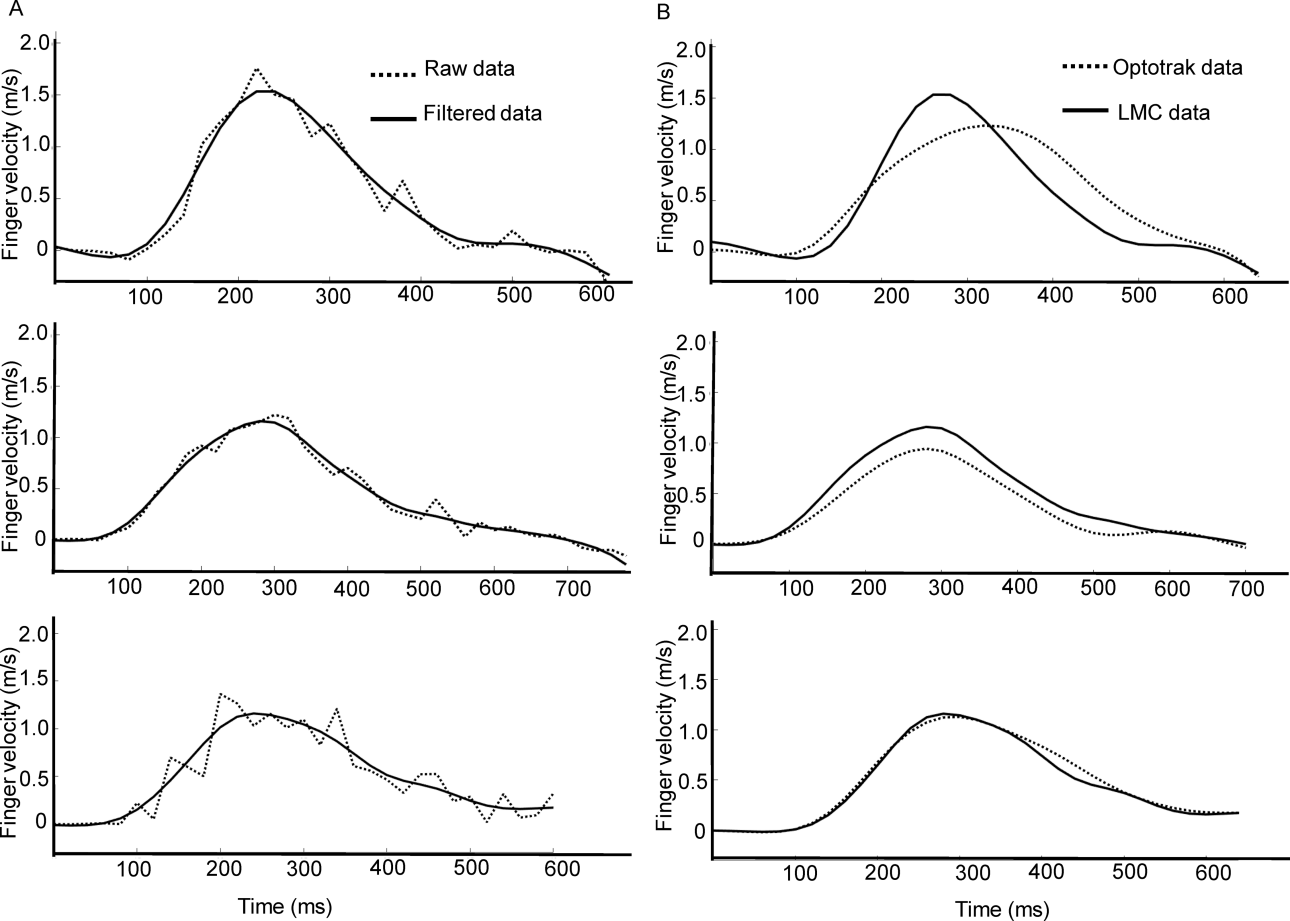
Three typical trials showing reach velocity data obtained along the primary direction of movement (i.e., depth axis) from the Optotrak and LMC. (A) Raw and filtered velocity data from the LMC trials that were accepted for analysis. (B) Comparison of the Optotrak and LMC velocity data obtained on concurrent trials.

The Optotrak and resampled LMC position data were filtered using a dual low-pass, second order Butterworth filter with a cut off frequency of 6Hz. Hand velocity was computed using a 2-point differentiation method, where instantaneous velocities were calculated using adjacent points. All Optotrak and LMC trials, including position and velocity traces, were screened by one of the authors for missing frames and noise artifacts. Table 1 provides a summary of the total number of trials that were included in the analysis for each participant. On average, less than 5% of trials from the Optotrak were excluded due to marker occlusion (>40 ms, which represents 2 missing frames) during the reaching movement. Fig 2 shows the raw and filtered velocity data for 3 trials recorded with the LMC. Visual inspection of each trial revealed a high-frequency artifact in the LMC velocity data, and these trials (~10% of the data) were excluded from further analysis. Fig 3A shows the raw and filtered velocity data for 3 typical trials recorded with the LMC system. The filter effectively removed high frequency noise from the velocity data, and these trials were accepted for further analysis. The filtered LMC and corresponding Optotrak data are plotted in Fig 3B, which shows that the LMC typically overestimates peak velocity when compared to the Optotrak.

Reaching performance was quantified by calculating movement duration, the duration of the acceleration and deceleration interval, as well as peak velocity. Although not the focus of the current study, endpoint error, which is a measure of spatial accuracy, was also examined. The same custom made Matlab script was used to analyse the data recorded with the Optotrak and LMC systems. Movement duration was defined based on a velocity criterion obtained from the index finger data: 20 mm/s for movement initiation, and 100 mm/s for defining the end of the reach. These criteria are consistent with previous literature [5,19–21]. Duration of the acceleration phase was the time interval from movement initiation to reach peak velocity, and the duration of the deceleration phase was the time interval between time of peak velocity and the end of movement. Peak velocity was defined as the maximum value obtained from the velocity data in the depth axis. Endpoint accuracy was defined as the distance along azimuth between the target and the index finger at the end of the movement. Precision error was defined as the within subject standard deviation across the reaching trials for a particular target location. Validity (bias) and limits of agreement between the kinematic measures obtained from the LMC and the Optotrak were examined using a Bland-Altman test. One sample t-test was also conducted to test the null hypothesis that there would be no significant difference between the measures obtained from the Optotrak and LMC.

### Results

**Fig 4:**
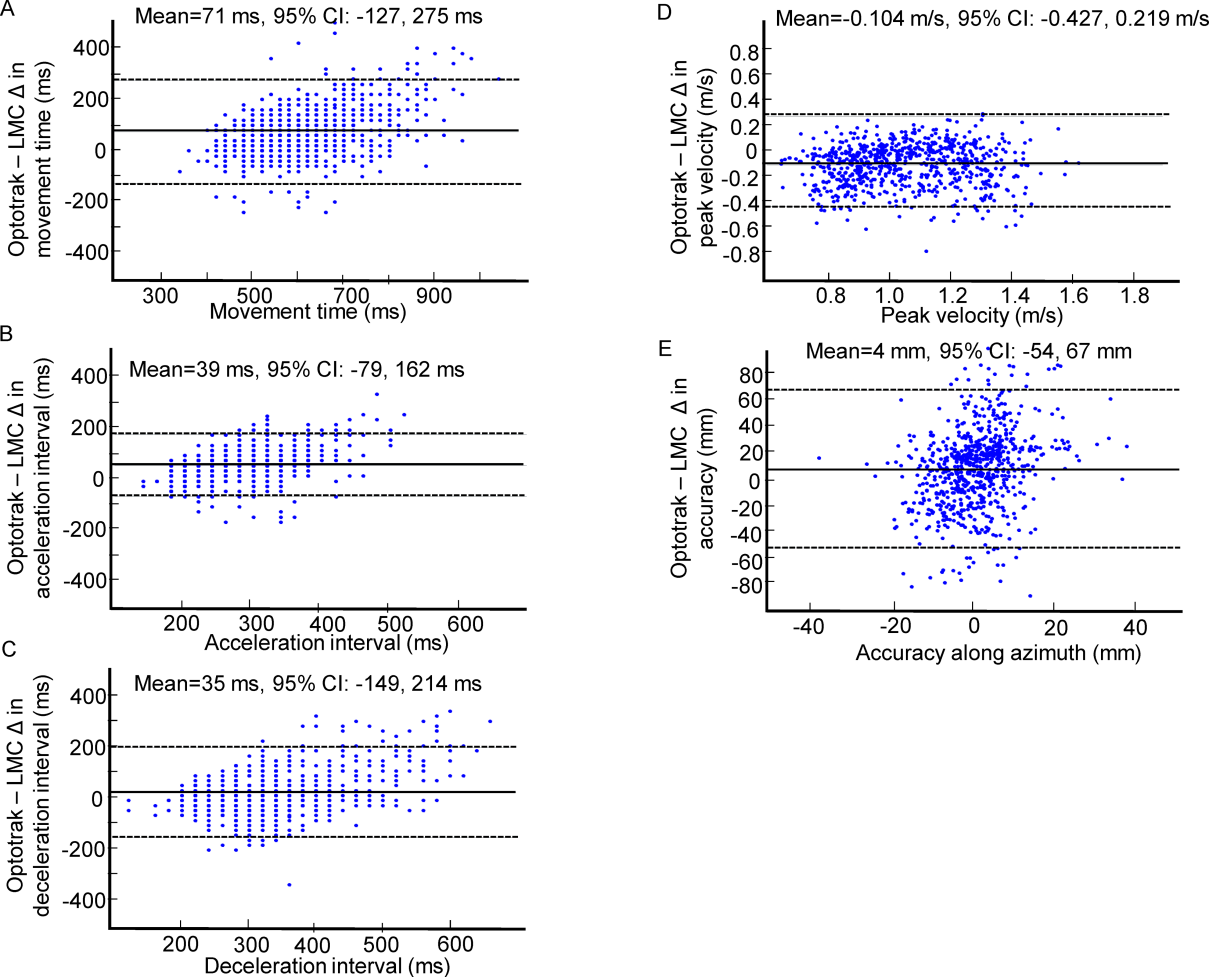
Bland-Altman plots for kinematic outcome measures. Difference (Δ Optotrak–LMC) in (A) movement time, (B) duration of the acceleration interval, (C) duration of the deceleration interval, (D) peak velocity, (E) endpoint accuracy along azimuth.

**Fig 5:**
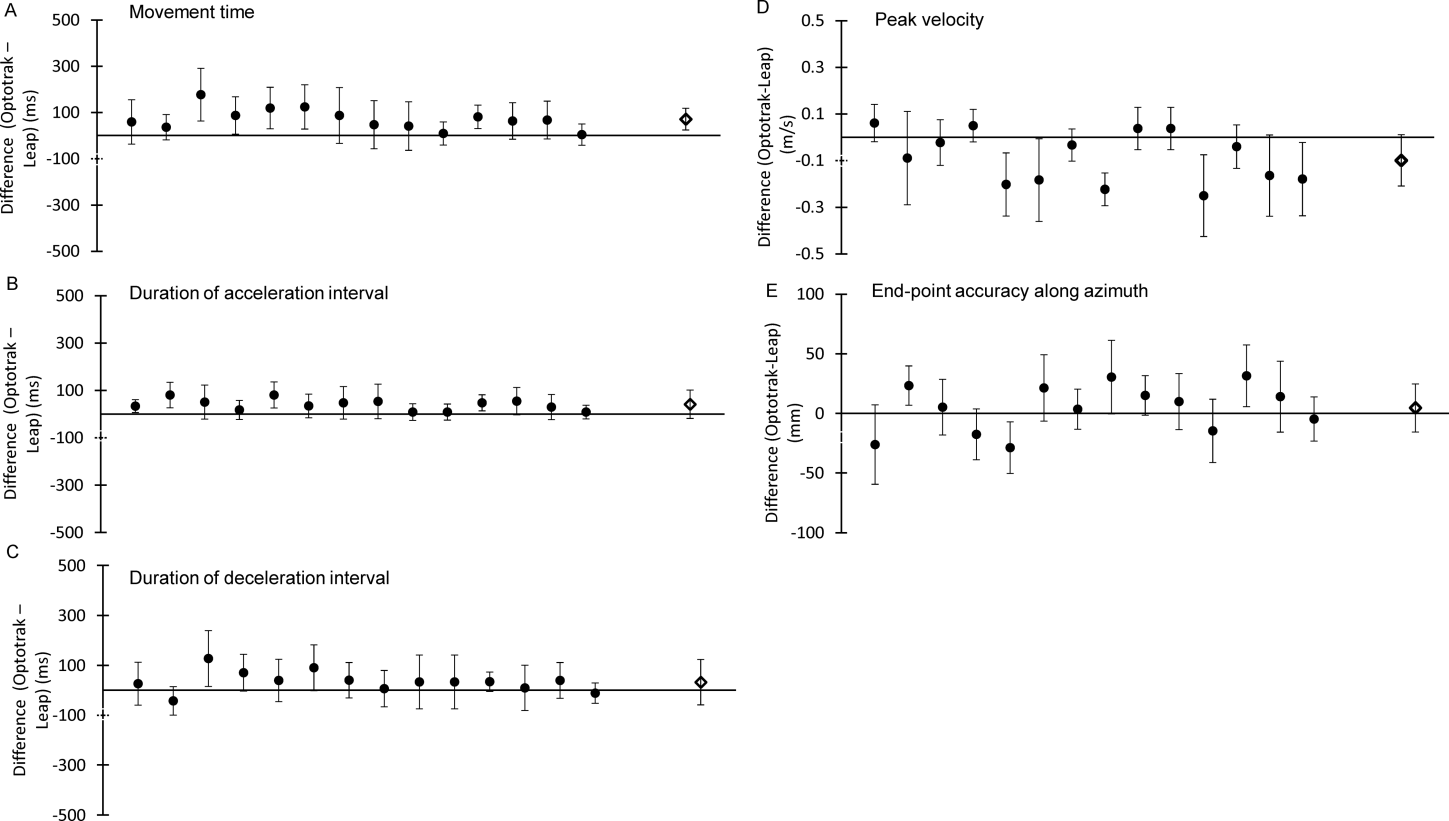
The average difference between the Optotrak and LMC, and the associated standard deviation for each outcome measure plotted for participants 1–14. (A) movement time, (B) duration of the acceleration interval, (C) duration of the deceleration interval, (D) peak velocity, (E) endpoint accuracy along the azimuth. Each filled circle represents the mean±standard deviation. The unfilled diamond symbol represent the grand average and the corresponding standard deviation.

Fig 4 shows the distribution of differences (i.e., errors) in kinematic outcome measures between the Optotrak and LMC across all trials (n = 728, see S1 Table for results from individual participants). The 3 temporal measures were movement duration, duration of the acceleration interval, and duration of the deceleration interval, as well as one spatiotemporal measure: peak velocity. Although our main goal was to assess the temporal and peak velocity accuracy of the LMC system, limb position at the end of the movement was also examined. The difference in outcome measures between the 2 systems (Optotrak–LMC) on each trial is plotted on the y-axis, while the x-axis shows the values obtained with the Optotrak, with positive values indicating that the LMC underestimates the measured outcome. Typically, the Bland-Altman analysis involves plotting the average scores obtained from the two devices on the x-axis because it is expected that both devices can contribute to measurement error. However, in the case of devices used in this study, the Optotrak is the criterion-standard for recording kinematic data, with validated submillimeter spatial precision and reliable (user-specified) sampling frequency. Bland-Altman analyses were also conducted separately for each participant to obtain the average difference between the Optotrak and LMC, and the associated confidence interval. Fig 5 illustrates the average differences and the associated standard deviation for all outcome measures obtained from each of the 14 participants, as well as the grand average calculated across all the participants.

#### Temporal accuracy

As shown in Fig 5A, the LMC system tends to underestimate movement duration. The grand average difference in movement duration was 71±47 ms. Although the difference in movement duration was <100 ms for 11/14 participants, the maximum difference for one of the participants was 177 ms. Results from the one-sample t-test showed that the error in movement time was significantly different from zero (t_13_ = 5.60, p<0.0001).

The duration of the acceleration interval was underestimated by the LMC system by 39±24 ms (Fig 5B). For 10/14 participants the difference between the Optotrak and LMC was <50 ms. Similarly, the duration of deceleration interval was underestimated by the LMC system by 35±42 ms (Fig 5C), and for 11/14 participants this error difference was <50 ms. Results from the one-sample t-test showed that the errors in the duration of acceleration and deceleration intervals were significantly different from zero (t_13_ = 6.07, p<0.0001, t_13_ = 2.73, p = 0.017, respectively).

#### Peak velocity

The grand average difference between the Optotrak and LMC system for peak velocity was 0.104±0.112 m/s (Fig 5D). Although the difference in peak velocity was <0.100 m/s for 9 of the 14 participants, for one of the participants the difference was 0.251±0.175 m/s. Results from the one-sample t-test showed that the error in peak velocity was significantly different from zero (t_13_ = -3.51, p = 0.004).

#### Spatial accuracy & precision

The grand average difference between the Optotrak and LMC systems in endpoint accuracy along the azimuth was +4.52±19.94 mm (Fig 5E). The mean error and standard deviation for each target location was +2.55±30.29 mm for the -10° target, +14.90 ±28.75 mm for the -5° target, +0.58±22.91 mm for the 5° target, and +4.00±17.22 mm for the 10° target. Examining the average differences from the individual participants showed that the maximum and minimum values were 28.81 mm (undershoot error) and 31.84 mm (overshoot error), respectively. The limits of agreement for each participant were also wide, ranging between ±16.78 mm to ±33.28 mm. Due to the large variability that included both undershoot and overshoot errors, results from the one-sample t-test showed that the error in endpoint accuracy was not significantly different from zero (t_13_ = 0.82, p = 0.429).

Endpoint spatial precision was calculated as the within-subject standard deviation across trials performed to targets at the same location. The mean error in endpoint precision was 10.43±5.55 mm, across the participants the error ranged between 3.85 to 21.30 mm. Results from the one-sample t-test showed that the error in endpoint precision was significantly different from zero (t_13_ = -7.03, p<0.0001).

### Discussion

To our knowledge, this study is the first to examine the temporal accuracy of the LMC system in visually-guided reaching tasks. Results showed that the LMC system tends to underestimate the duration of reaching by 71±47 ms, which represents a ~13% error of total movement time. Only one of 14 participants in our sample exhibited a large difference in movement time (177ms) when the LMC was compared to the Optotrak. Interestingly, this underestimation of movement time was not associated with increased error difference in peak velocity, which was only 0.020 m/s for this participant. On average, the acceleration and deceleration intervals were also underestimated by 39±24 ms and 35±42 ms, respectively.

The average peak velocity obtained with the LMC system was overestimated by 0.104±0.112 m/s, which represents ~10% error compared to values obtained with the Optotrak system. Because the sampling rate of the LMC cannot be set by the user, we investigated whether this underestimation error is related to sampling frequency. Examining Table 1 and Fig 5 closely revealed no consistent relationship between sampling frequency and underestimation error. For example, P5, P6, and P8 were among the participants with the largest differences in peak velocity between the LMC and the Optotrak and the highest sampling rate. Conversely, the sampling rate for P10 was only 56Hz, but the peak velocity error was low. We also considered the possibility that the reliability of the LMC system in measuring peak velocity is dependent on movement speed. To examine this, a Pearson’s correlation coefficient was computed to assess the association between the Optotrak-LMC difference in peak velocity (i.e., error) and reach peak velocity as recorded by the Optotrak. This analysis showed a weak negative association (r = -0.17), which was not statistically significant, and does not support a relation between error and the magnitude of peak velocity (as measured by the Optotrak). Therefore, it is not clear what conditions contribute to greater peak velocity error, and this question needs to be examined further in future studies.

Results from the first experiment showed that the spatial accuracy measures can not be obtained reliably from the LMC system. These results are consistent with the study by Tung and colleagues which examined the spatial accuracy obtained with the LMC and Optotrak when participants pointed to a visual target displayed on a computer monitor [17]. Their results showed high correlations for limb position obtained with the LMC and Optotrak; however, the limits of agreement were wide, which indicates that the trial-to-trial estimates of limb position obtained from the LMC system were highly variable. In the current study, the spatial accuracy across participants ranged between -28 mm to +31 mm, and within-participant precision (i.e., standard deviation) was up to 30 mm. These results are in contrast to previous studies that used a robotic device or a plastic arm model and reported high spatial accuracy (<5 mm) for the LMC system [14,15]. The main difference between these studies and our results is that we assessed young adults, and the accuracy and precision were obtained at the end of a natural movement rather than during a static hold position or dynamic trials conducted with a robotic device.

### Experiment #2: Speed-accuracy trade-off

Considering that the temporal measures obtained with the LMC system appear to be more reliable compared to the spatial measures, the second experiment was conducted to assess whether the LMC can be used to detect the speed-accuracy trade-off (i.e., Fitts’ law). The relation between movement speed and endpoint accuracy is one of the fundamental laws in movement control: movements that require accuracy and precision (i.e., when the target is small) have longer movement times [22], and more specifically, it is the deceleration phase of the movement that is extended [23]. When stressing endpoint accuracy in the instructions and presenting targets of different sizes, Fitts’ Law would predict that movements towards smaller targets will have longer movement times, and a longer deceleration phase. It was hypothesized that the LMC system will provide a reliable estimate of the speed accuracy trade-off. More specifically, it was expected that movement time and deceleration phase will be significantly longer when moving towards small targets. Because the results from experiment 1 showed that the LMC does not provide reliable measures of spatial accuracy, endpoint error was not examined in experiment 2.

Peak velocity is an important kinematic measure that provides insight into movement planning [24,25]; therefore, the efficacy of the LMC system to measure peak velocity was further examined in experiment 2. Previous studies have shown that aiming movements to the ipsilateral side (i.e., rightward movement for the right hand) have a larger peak velocity compared to movements directed to contralateral targets [26,27]. In the current study, targets were presented 10 degrees to the left and right of participant’s midline, which allowed us to examine the effect of target location on peak velocity. It was hypothesized that the LMC system will provide a reliable estimate of peak velocity and discriminate between movements to the ipsi- and contralateral targets.

#### Participants

Fifteen young adults (23±5 years; 8 females) recruited from the University of Waterloo participated. Two of the participants also participated in experiment 1. All participants had normal visual acuity (20/20), and reported to be right-hand dominant. None of the participants reported any neuromuscular symptoms, or previous injuries to the upper limb.

#### Apparatus

The same experimental set-up was used as in experiment 1. The stimulus for visually-guided aiming was a white circle with four different sizes (0.25°, 1.0°, 2.5°, 5°), which was presented at 2 locations along the azimuth (±10°). Each stimulus was presented 20 times (total of 80 trials per participant), and the order of presentation was randomized, which was controlled by a custom VPixx script (VPixx Technologies, Quebec, Canada).

#### Procedure

The protocol was similar to experiment 1, except participants were instructed to prioritize accuracy over speed.

#### Data processing

Data were analysed offline using a custom Matlab script (Mathworks Inc, Natick, MA, USA). Data processing involved the same procedures as described in the Methods-Data Processing section in experiment 1. To examine the speed-accuracy trade-off, the analysis focused on comparing two temporal measures (movement time, duration of deceleration interval). Peak velocity was used to examine the effect of target location. A repeated-measures analysis of variance (ANOVA) was conducted separately for the Optotrak and LMC datasets to determine whether the effect of target size and target location leads to comparable statistical results when using the LMC system.

### Results

**Fig 6:**
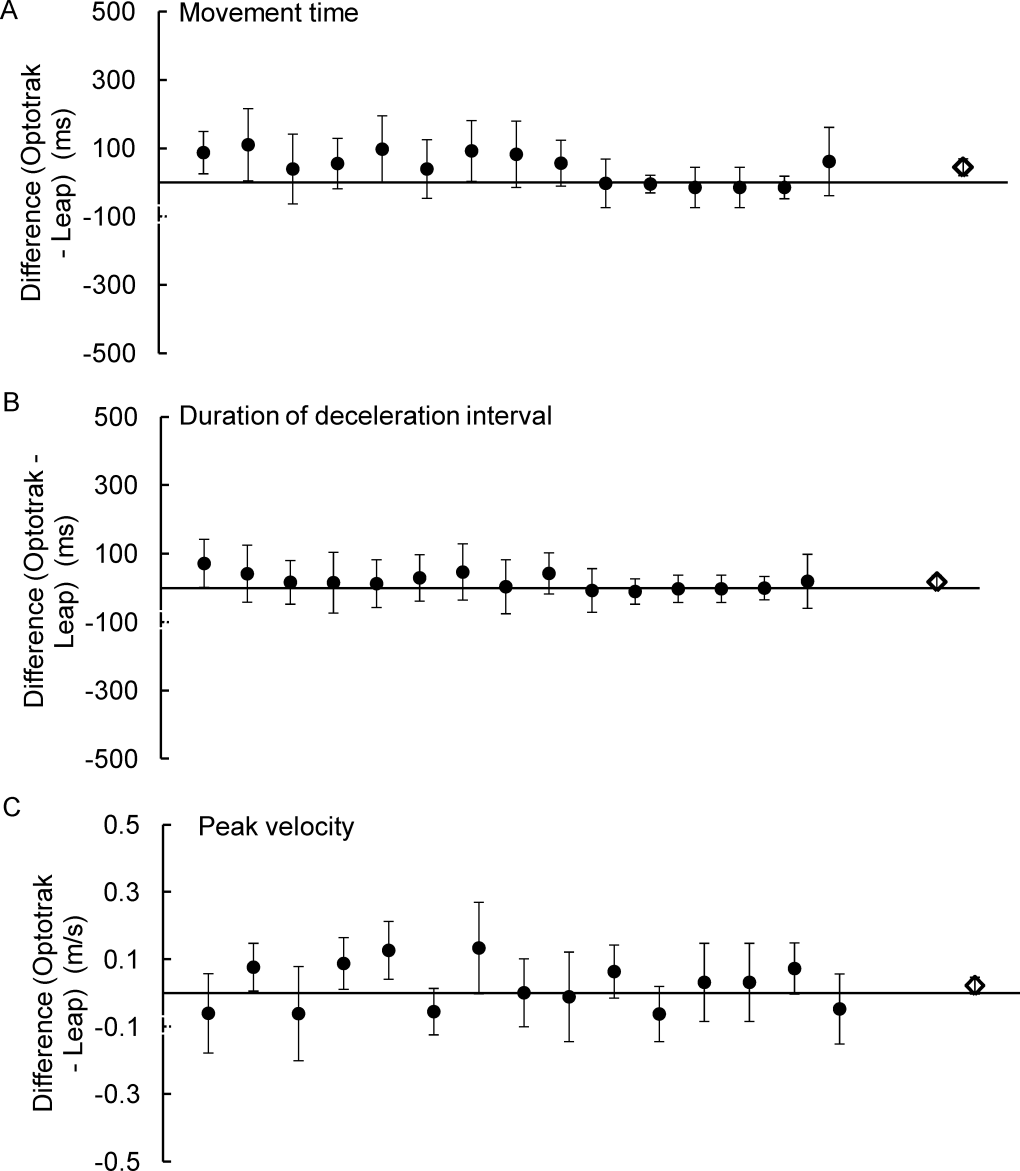
The average difference between measures obtained from the Optotrak and LMC systems, and the associated standard deviation plotted for each of the 15 participants. (A) movement time, (B) duration of the deceleration interval, (C) peak velocity. Each filled circle represents the mean±standard deviation. The unfilled diamond symbol represented the grand average and the corresponding standard deviation.

Fig 6 shows the mean differences between measures obtained from the Optotrak and LMC for each participant (see S2 Table for results from individual participants). Similar to the results from experiment 1, movement time was underestimated by the LMC: the mean movement time difference was 44±25ms (~10% error of total movement time). Across the 15 participants, the differences in movement time ranged between 15 ms (overestimation) to 110 ms (underestimation). Results from the one-sample t-test showed that the error in movement time was significantly different from zero (t_14_ = 4.29, p = 0.0008). The mean difference in duration of the deceleration interval was 19±18ms, and the range across the participants was 11 ms (overestimation) to 71 ms (underestimation). Results from the one-sample t-test showed that the error in deceleration interval was significantly different from zero (t_14_ = 3.29, p = 0.005). Lastly, the mean difference in peak velocity was 0.021±0.025 m/s, with 13/15 participants showing a difference <0.100 m/s. As shown in Fig 6 the within-subject standard deviation was as high as ±0.133 m/s in some participants. Results from the one-sample t-test showed that the error in peak velocity was not statistically different from zero (t_14_ = 1.39, p = 0.189).

The association between peak velocity error and the peak velocity measured by Optotrak was also examined in experiment 2. A Pearson’s correlation coefficient was calculated for each participant’s data. Results showed a low to moderate association across the participant sample. The average Pearson’s correlation coefficient was 0.24±0.19, range: -0.10 to 0.49).

**Fig 7:**
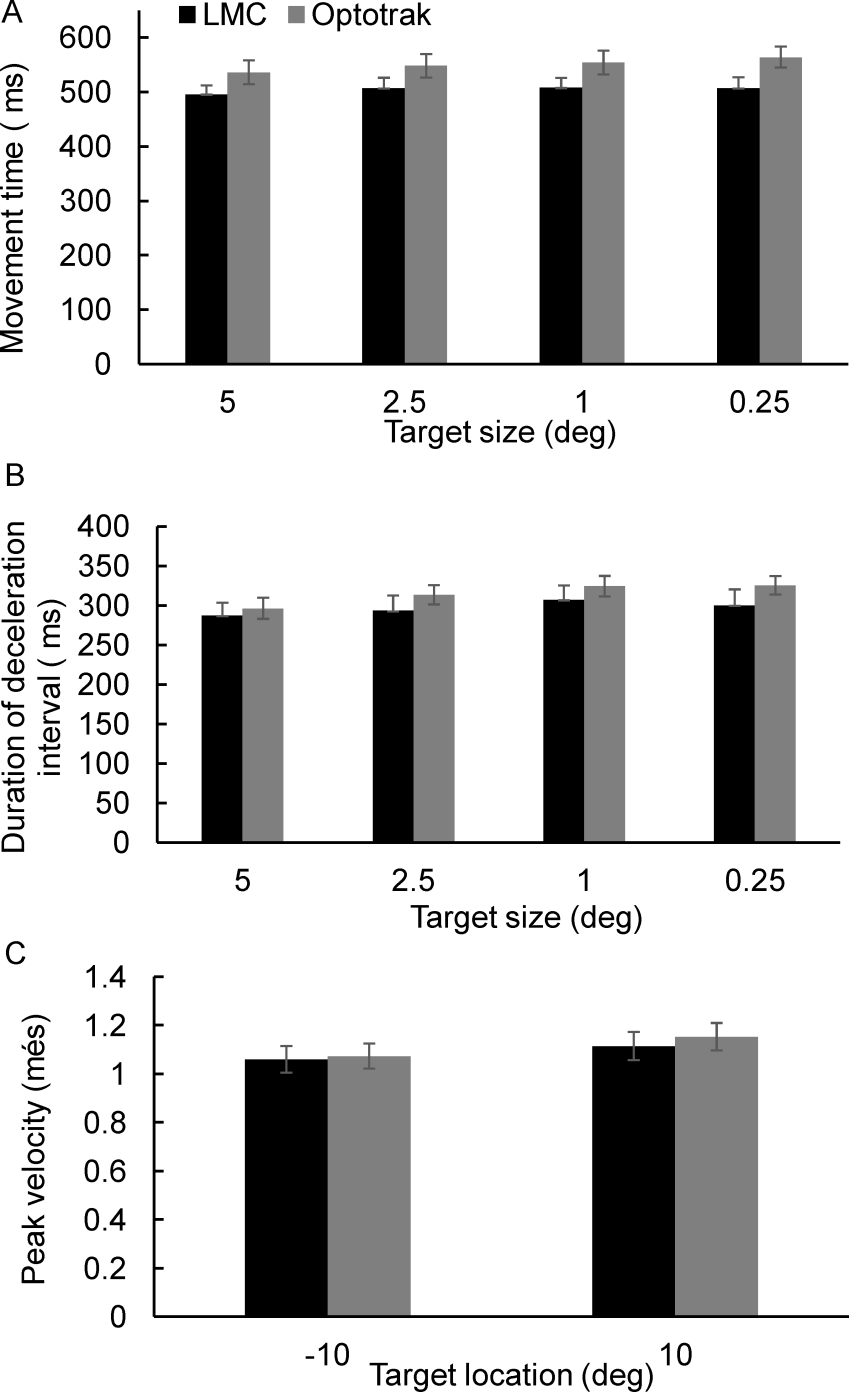
Grand average obtained from the 15 participants. (A) movement time, (B) duration of the deceleration interval, (C) peak velocity. Error bars show standard error of the mean.

Results from the ANOVA for the Optotrak data showed a clear speed-accuracy trade-off for both movement time (F_3,42_ = 4.93, p = 0.005, η^2^ = 0.19; Fig 7A), and duration of deceleration interval (F_3,42_ = 9.68, p<0.0001 η^2^ = 0.31; Fig 7B). The speed-accuracy relationship was only evident in the LMC dataset for the duration of the deceleration interval (F_3,42_ = 3.33, p = 0.029, η^2^ = 0.14; Fig 7B), but not for movement time (F_3,42_ = 1.24, *ns*; Fig 7A). Analysis of variance showed that the effect of target location on peak velocity was statistically significant for both, the Optotrak data (F_1,14_ = 17.13, p = 0.001, η^2^ = 0.71) and the LMC data (F_1,14_ = 5.11, p = 0.039, η^2^ = 0.42; Fig 7C).

### Discussion

The aim of experiment 2 was to assess whether the LMC system could be used to assess the speed-accuracy trade-off relationship, a fundamental law in motor control. Only one previous study compared the LMC system with a mouse-controller during the performance of a Fitts’ task [16]. They found large discrepancies in errors and movement duration (>300ms difference); however, collection with the LMC and the mouse-controller was not simultaneous. The current study extends these earlier findings by recording each trial concurrently with the LMC and Optotrak systems. Our results showed that movement time was underestimated by the LMC on average by 44 ms (i.e., ~10% error of total movement time); however, for one of the participants the error was 110 ms. The current study also provides more detail into the speed-accuracy trade-off by assessing the duration of deceleration interval, in addition to total movement time.

According to Fitts’ law, movement speed must be adjusted to maintain accuracy when aiming towards smaller targets [22]. The reduction in target size is associated with a proportional increase in movement duration and more specifically, a longer deceleration interval [23]. Results from the second experiment partially supported our hypothesis that the LMC would provide a reliable estimate of the speed-accuracy trade-off. Data obtained from the LMC system provided evidence of speed-accuracy trade-off when considering the duration of deceleration phase, but not the total movement duration. The lack of sensitivity of the LMC system to discriminate the effect of target size using movement time is most likely due to greater within and between-subject variability (i.e., smaller effect size), and the error associated with the LMC measurement system. In contrast to total movement duration, the effect size obtained with the Optotrak system for the duration of deceleration interval was twice as large, and thus, despite the LMC’s inherent measurement error, the difference between conditions achieved statistical significance.

With regards to peak velocity, the average difference between the Optotrak and LMC systems across the participants ranged from 0.087 m/s (8% overestimation) to 0.133 m/s (12% underestimation). Despite these differences, our second hypothesis was also supported by the data obtained from the LMC: peak velocity was significantly higher when pointing to ipsilateral compared to contralateral targets. As shown in Fig 7, the mean difference in peak velocity between the ipsilateral and contralateral targets was 0.055 m/s for the LMC data (η^2^ = 0.42), and 0.080 m/s for the Optotrak data (η^2^ = 0.71), while the variance (i.e., standard deviation) in the two datasets was comparable. Considering the smaller effect sizes obtained when using the LMC system, it is very important to consider the expected effect size when using the LMC to collect kinematic data.

### Experiment #3: Reach, grasp, and place

The ability to perform prehension movements is fundamentally important for activities of daily living. Therefore, clinical assessment of upper limb movements often includes tasks involving reaching, grasping and manipulating objects of different shapes and sizes. Two tasks that are often used for assessment are the peg-board and bead-threading [28]. Previous work has shown that the bead-threading task is more difficult when compared to the peg-board because it involves grasping a smaller object, and greater precision is required to place the bead on the needle [29,30]. Furthermore, performance on the bead-threading task can be used to discriminate age-related improvements in upper limb function [29], and to detect deficits in motor control in people with abnormal binocular vision [30], as well as children with poor reading ability [31]. Therefore, the bead-threading task was used in the current study to examine how well the LMC system can measure the temporal aspects of the bead-threading sequence (i.e., the duration of reaching, grasping and placement), as well as peak velocity.

#### Participants

Fifteen young adults (22±3years; 7 females) recruited from the University of Waterloo participated. None of them participated in experiment 1 or 2. All participants had normal visual acuity (20/20), and reported to be right-hand dominant. None of the participants reported any neuromuscular symptoms, or previous injuries to the upper limb.

#### Apparatus

**Fig 8:**
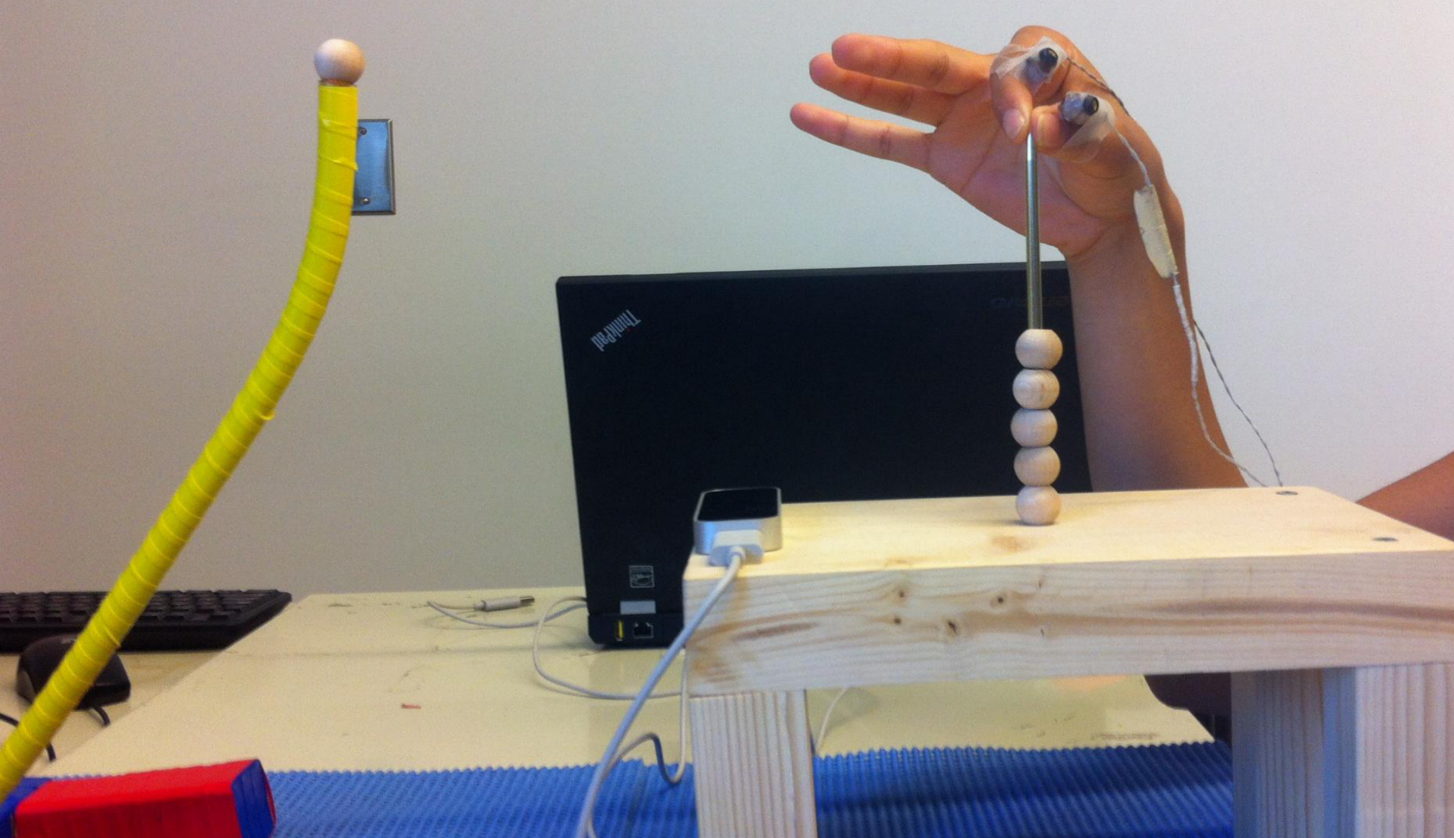
Picture illustrating the experimental set-up used in experiment 3.

The experimental set-up is illustrated in Fig 8. Participants performed the bead-threading task which consists of a reaching, grasping, and placement components. The IREDs were placed on the intermediate phalanx of the index finger and the proximal phalanx of the thumb. Each trial was initiated with the participant’s hand in a standardized position holding the tip of a blunt needle with their thumb and index finger. A small bead (diameter 16 mm) was placed 30 cm in front, and participants were asked to reach and grasp the bead, and then place it on the blunt needle as quickly as possible. Participants were asked to move their hand away from the needle when finished with the bead placement in order to have a clear marker of task completion. Because we were specifically interested in determining whether the LMC can be used to determine the duration of each of the components of the movement sequence, Optotrak data were sampled at 250Hz and the LMC data were upsampled to 250Hz in order to increase the temporal resolution.

#### Data processing

Data processing involved the same procedures as described in the Methods-Data Processing section in experiment 1. LMC position data were first fitted using a cubic spline function and resampled at 250 Hz (using matlab function pchip). Trials with more than 40 ms of missing data were excluded from further analysis. Data were analysed offline using a custom Matlab script (Mathworks Inc, Ntick, MA, USA). The main analysis focused on comparing the difference between the Optotrak and LMC measures (i.e., error) for the following components of a movement sequence: duration of reach to the bead (reach-to-bead), grasp time, duration of reach to the needle (reach-to-needle), placement time, as well as peak velocity when reaching to the bead (PV-bead) and to the needle (PV-needle). Reach duration was defined based on a velocity threshold: the time when finger velocity first reached >30mm/s for 20 consecutive milliseconds was used to detect movement initiation, and velocity <100 mm/s was used to define the end of the reach. Grasp time was defined as the interval from the end of reach-to-bead to the initiation of reach-to-needle. Placement time was defined from the end of reach-to-needle to the initiation of a reach away from the needle. These criteria are consistent with our previous work [19,20], as well as the aiming literature [6,32,33]. Because maximum grip aperture (MGA) is often used to assess grasping movements, MGA obtained from the Optotrak and the LMC systems was also compared during the bead-threading task. MGA was defined as the maximum separation between the index and thumb fingers during the reach-to-bead movement.

### Results

**Fig 9:**
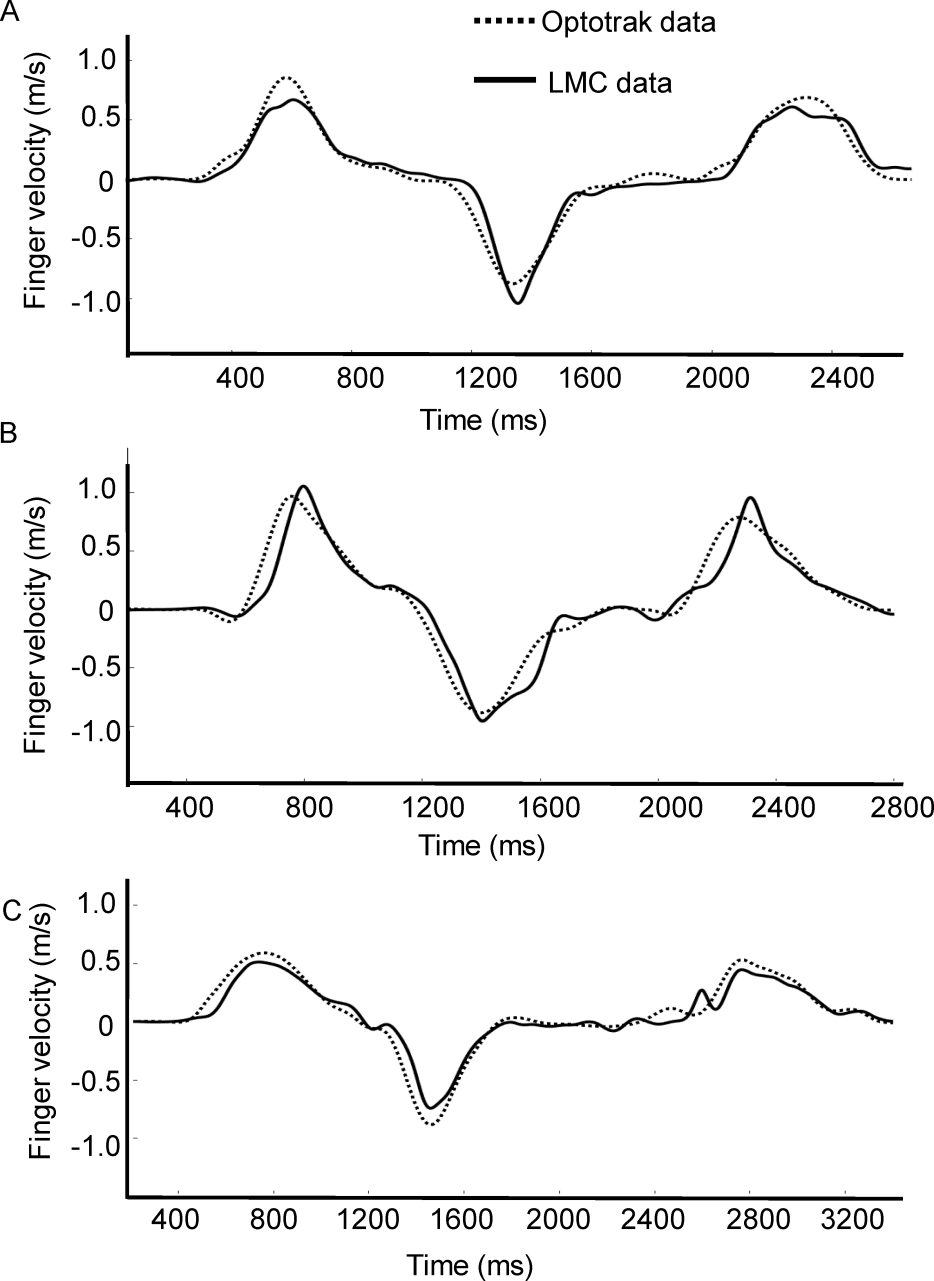
Three typical trials illustrating finger’s velocity during the bead threading task obtained from the Optotrak and LMC systems on concurrent trials.

**Fig 10:**
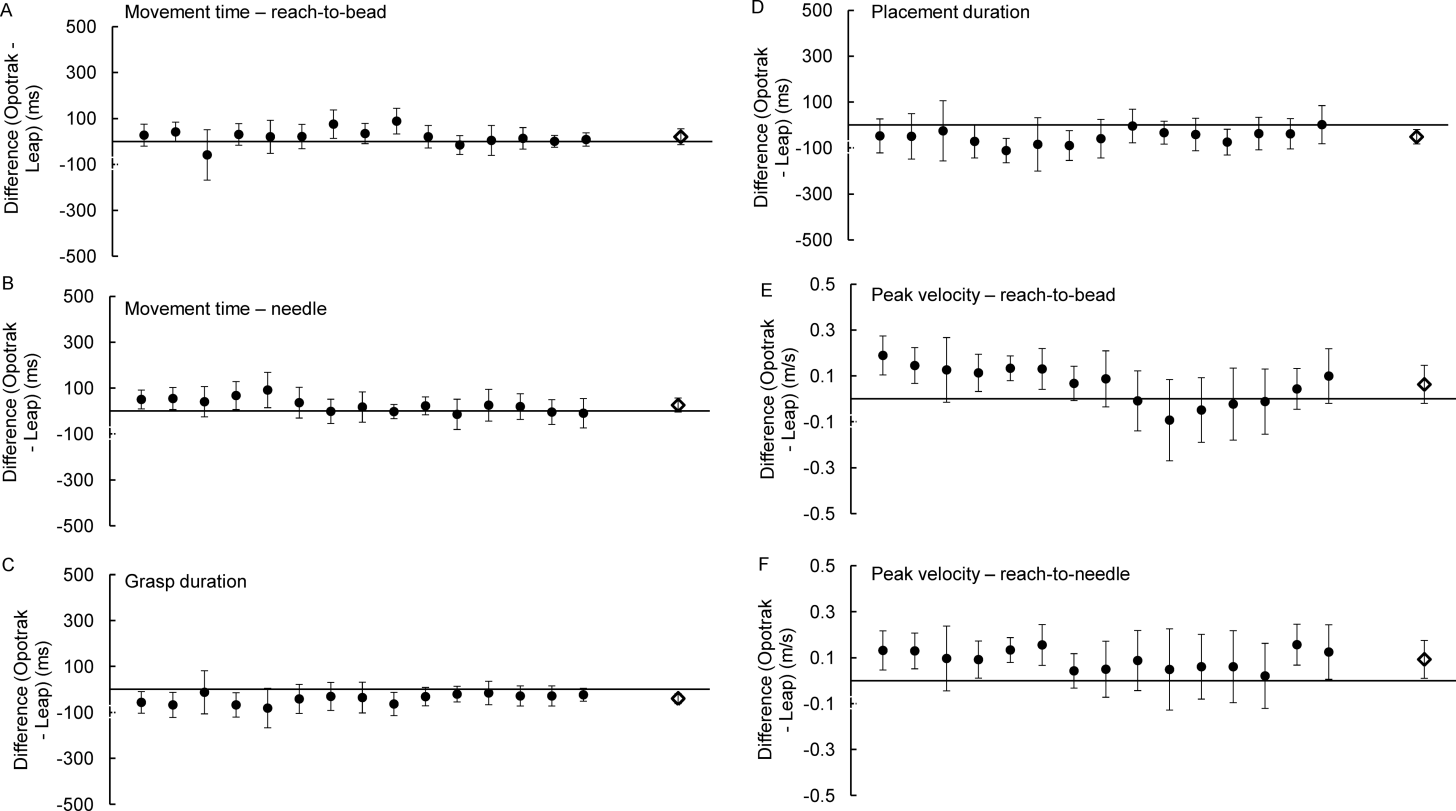
The average difference between measures obtained from the Optotrak and LMC systems, and the associated standard deviation plotted for each of the 15 participants. (A) reach-to-bead duration, (B) reach-to-needle duration, (C) grasp time, (D) placement time, (E) PV-reach-to-bead, (F) PV-reach-to-needle. The unfilled diamond symbol represents the grand average and the corresponding standard deviation.

**Fig 11:**
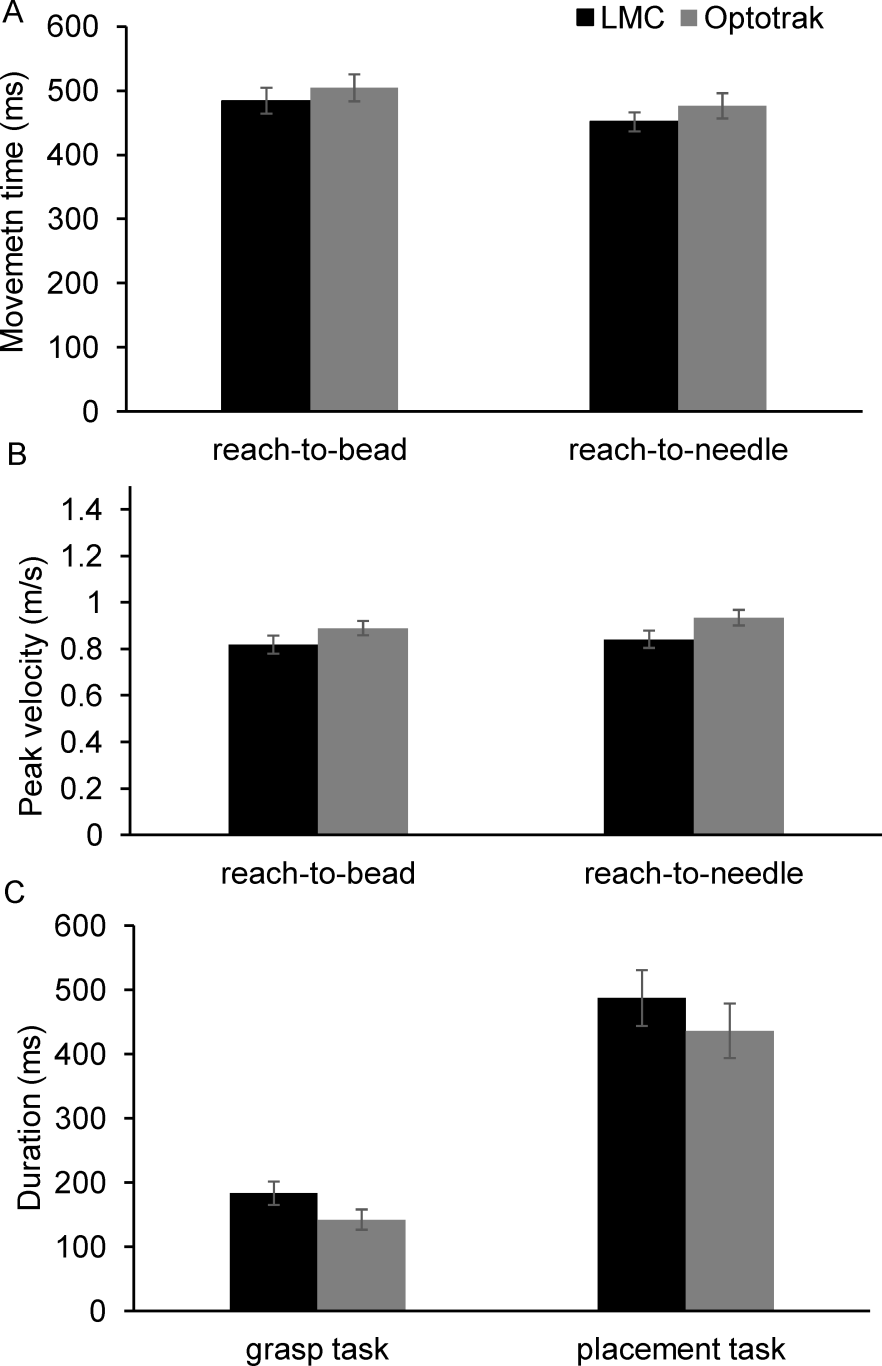
Grand average obtained from the 15 participants for each outcome measure. (A) movement duration for reach-to-bead and reach-to-needle, (B) PV-bead and PV-needle, (C) duration of the grasp and placement tasks. Error bars show standard error of the mean.

Fig 9 shows limb velocity obtained from the Optotrak and LMC systems on 3 concurrent trials. Mean differences between the measures obtained from the Optotrak and LMC for each participant are shown in Fig 10 (see S3 Table for results from individual participants). The mean reach-to-bead and reach-to-place durations were underestimated by the LMC system by 20±35 ms (t_14_ = 2.27, p = 0.039) and 26±31 ms (t_14_ = 3.24, p = 0.006), respectively. This represents ~5% error of the reach movement time. The LMC system overestimated the duration of grasping time (41±22 ms, 30% error; [t_14_ = -7.27, p<0.001]) and placement time (52±31 ms, 11% error; [t_14_ = -6.42, p<0.001]). In contrast to the results from the first two experiments, the average peak velocity was underestimated by the LMC: PV-bead was 0.063±0.083 m/s (range: -0.093 [overestimation] to 0.189 [underestimation]; [t_14_ = 3.51, p = 0.003]); PV-needle was 0.092±0.044 m/s (range: 0.02 to 0.156 [underestimation]; [t_14_ = 8.08, p<0.0.0001]). Finally, the average difference in MGA was 11±11 mm (across participants ranged -9 to 33 mm; [t_14_ = 3.76, p = 0.002]), while the largest standard deviation for one of the participants was ±29 mm. The means for all kinematic measures obtained with the Optotrak and LMC systems are plotted in Fig 11.

### Discussion

The goal of experiment 3 was to evaluate the capability of the LMC system to assess the temporal accuracy and peak velocity during the performance of a movement sequence involving reaching, grasping, and placing components. The bead-threading task was selected because previous studies have shown that it might be sensitive for discriminating changes in motor control during normal development, as well as deficits in motor control in adults and children [29,30]. Performance on the bead-threading task in a clinical setting is usually evaluated using a stopwatch. However, using the LMC could provide a greater insight into motor performance, and to identify which components of the movement sequence are affected. The advantage of using a system such as LMC in comparison to a stopwatch is that movement components are identified consistently across trials based on velocity criteria. Our results show that the LMC system provides robust estimates of temporal measures, such as duration of the reach and placement (average error 5% and 11%, respectively). In contrast, the grasp duration was associated with a larger error, ~30%, which is most likely due to the fact that grasp duration was shorter compared to the placement and reach components (142 ms vs 436 ms vs 500 ms). In contrast to the aiming experiments 1 and 2, average peak velocity was underestimated by the LMC system when reaching towards the bead or the needle by ~10%. Despite the bias/error, the results obtained with the LMC system were in agreement with the Optotrak results: average peak velocity was higher and movement time was longer for reaches to the needle compared to reaches to the bead.

Maximum grip aperture (MGA) scales precisely with object size, therefore, it is an important measure in prehension studies reflecting the participant’s ability to perceive object size. Because MGA is a spatial measure based on the distance between the index finger and the thumb, our results showed that the LMC’s accuracy for measuring MGA is similar to the spatial accuracy of the finger during performance of the aiming movement in experiment 1.

## General discussion

The objective of this study was to evaluate the reliability and validity of the LMC system for measuring movement kinematics as compared to a criterion-standard motion capture system, the Optotrak. Specifically, our main goal was to assess temporal accuracy and peak velocity during the performance of visually-guided upper limb movements. Table 2 provides a summary of the mean difference error between the Optotrak and LMC data for outcome measures assessed in experiments 1–3. In the following general discussion we will first address the technical limitations, and then consider the suitability of the LMC system to assess upper limb function.

**Table 2:** Summary of the mean difference error (Optotrak-LMC) in outcome measures found in experiments 1–3.

Difference (Optotrak-LMC)	Experiment 1	Experiment 2	Experiment 3
Movement time (ms)	71± 47	44±25	Reach-to-bead: 20±35 Reach-to-needle: 26±31
Duration of deceleration (ms)	35±42	19±18	NA
Peak velocity (m/s)	-0.104±0.112	0.021±0.025	PV-bead: 0.063±0.083 PV-needle: 0.092±0.044
Grasp time (ms)	NA	NA	-41±22
Placement time (ms)	NA	NA	-52±31
Endpoint accuracy (mm)	+4.52± 19.94	NA	NA

### Technical considerations

One of the main limitations of the LMC system is a lack of control over sampling rate, resulting in a variable sampling rate within and between trials. In this study the sampling rate ranged between 50 to >100Hz, which is sufficient to capture reaching and grasping movements [34]. Although the variable sampling rate has been suggested as a main factor limiting LMC’s usage [15], signal processing methods such as interpolation can be applied to reconstruct the data [35]. In the current study, an interpolation approach described by Tung et al. (2015) was applied. Specifically, the position data were fitted with a cubic spline and resampled at a constant rate. The raw and resampled data were visually inspected by the experimenter to ensure that this approach is valid and did not introduce artifacts, which was confirmed. A similar interpolation approach has been used recently for processing data acquired with the Kinect system, which has an even lower average sampling rate (i.e., ~30 Hz) [36]. In summary, signal reconstruction may be a viable approach that could be applied prior to further data processing, such as filtering or differentiation to obtain velocity.

Limitations associated with the spatial accuracy of the LMC system have been discussed in prior studies [17,37]. Although not the focus of the current investigation, our results clearly corroborate these studies: endpoint limb position during aiming movements, and MGA obtained during the grasping task were both highly variable. The LMC system uses a proprietary software to estimate the 3D position of the fingers, it is possible that improvements to the tracking algorithm in the future iteration of the software could improve the spatial accuracy measures. However, at the present time spatial accuracy for aiming movements should be obtained using other devices, for example, touch screens.

Our experiments were designed such that limb movements were performed in the volume optimal for LMC’s recording [15,17], and using a hand posture that maximized the number of valid trials [18]. Despite these experimental controls, the number of viable trials that were obtained from the LMC system was ~70% (range 40–90%). In contrast, on average only 5% of Optotrak data were excluded due to marker obstruction during the movement. This raises an important question that needs to be considered carefully: how many trials need to be collected to obtain a reliable estimate of the kinematics from the LMC system? This is particularly important given that the limits of agreement between the LMC and Optotrak were wide. In the current study, the mean for kinematic measures for each participant was calculated using at least 8 trials. Future studies could address this question directly by performing resampling experiments (e.g., Monte Carlo sampling).

#### Application to assessment of motor control

The ability to make skillful reaching movements depends on two sensorimotor processes: motor planning and feedback control [38–40]. Movement duration and the deceleration interval have been used in previous studies to infer feedback control [9]. For example, movements are longer when the task requires high accuracy and precision. Furthermore, availability of sensory feedback also modulates the degree of feedback control and trajectory adjustments in the latter part of the movement. Variability is an ubiquitous characteristic of human motor performance [41–43]. Typically, the within and between-subject standard deviation of movement duration for aiming movements ranges between 50–100 ms [44,45]. Therefore, we will use these criteria to interpret the utility of the LMC for estimating temporal measures. The range in mean movement duration found across the 3 experiments in the current study (~500–650 ms) is comparable to previous studies where the targets were presented at a distance of 40–50 cm [44,45]. Our results showed that average movement time error was <100 ms in 40/44 participants (93% of total sample), while error <50 ms was found in 66% of the sample. Therefore, for movements with duration of 500–600 ms the LMC temporal error could be in the range of 10–20% of total duration. The mean deceleration interval ranged between 300–350 ms, thus, a 50 ms difference would represent ~16% temporal error. Overall, the results from the 3 experiments reported here indicate that the LMC system provides robust temporal estimates of reaching movements; however, it is important to consider the limitations when using the LMC system. Specifically, we would not recommend using the LMC system in studies that focus on movements of short duration, and/or where the expected effect between groups or conditions is less than 100 ms.

Peak velocity is a kinematic measure that is closely associated with motor planning [46]. For example, peak velocity is modulated with reaching distance (i.e., higher peak velocity when reaching to farther objects), and the difficulty of a task (lower peak velocity when reaching to fragile objects). Across the 3 experiments, only 3 of the participants (5% of the sample) demonstrated a large error in average reach peak velocity, which was greater than 0.200 m/s, while 16 participants (27% of the sample) had errors in the range between 0.100 and 0.200 m/s. Therefore, the LMC system provided a robust estimate of peak velocity (i.e., error <0.100 m/s) for the majority of participants. It is possible that peak velocity estimates obtained with the LMC system could be used as a proxy measure to assess reach planning for aiming movements; however, only in studies where the expected effect size large.

One application where the LMC could provide a reliable measure of motor planning is using an experimental paradigm involving reaches to targets presented at different distances because peak velocity scales with movement amplitude. For example, MacKenzie and colleagues showed that peak velocity was 1.47 m/s for movement amplitude of 30 cm, and 0.92 m/s when the amplitude was 15 cm [23]. The LMC could also provide useful information when examining the developmental trajectory of upper limb reach control because changes in peak velocity indicate improvements in motor planning, which are associated with age. For example, Hay and colleagues reported that peak velocity increased from 0.75 m/s to 1.15 m/s when visually-normal children between the ages of 6–10 years performed a 30 cm aiming task [11]. Similar pattern of improvement in motor planning was reported for a reciprocal aiming task where the required movement amplitude was 24 cm: peak velocity was 0.62 m/s in a group 7 years old children, and 0.91 m/s in a group 11 years old children [47]. To summarize, previous literature indicates that the effect size for changes in peak velocity during development is large enough such that the LMC could be used to evaluate changes in motor control.

#### Conclusion

To summarize, this study was conducted to assess the capability of the LMC system to assess visually-guided upper limb movements. Our results indicate that the LMC provides robust estimates of temporal measures, such as movement time and the duration of deceleration interval. Our results also demonstrate that peak velocity obtained from the LMC could be used to assess motor planning, if the expected effect size is large. In contrast, spatial measures, such as endpoint accuracy, precision, and maximum grip aperture cannot be obtained reliably from the LMC system, therefore, we do not recommend using these measures.

The major advantage of the LMC is that it is a markerless, low-cost, highly portable system, which could facilitate collection of kinematic data outside of the traditional laboratory settings, such as clinics and schools. Because kinematic assessment provides insight into the control processes involved in movement planning and execution, the LMC could be used to improve the assessment of motor control during development, aging, and in people with musculoskeletal and neuropathological disorders, including stroke and concussions.

## Supporting information

S1 Table Experiment 1 results. Individual subject data averaged across the experimental conditions.
(PDF)

S2 Table Experiment 2 results. Individual subject data averaged across the experimental conditions.
(PDF)

S3 Table Experiment 3 results. Individual subject data averaged across the experimental conditions.
(PDF)
