# Supplementary material for: Evaluation of the Leap Motion Controller during the performance of visually-guided upper limb movements
Source: PLoS One. 2018 Mar 12;13(3):e0193639. doi: 10.1371/journal.pone.0193639 (PMC5846796; doi:10.1371/journal.pone.0193639)
Supplement: S1 Table — Individual subject data averaged across the experimental conditions. (PDF) [file pone.0193639.s001.pdf]

		Movement time (s)					
		Optotrak		LMC		Difference	
Participant	Location	Mean	Std Dev	Mean	Std Dev	Mean	Std Dev
1	-10	0.635	0.066	0.505	0.046	0.130	0.076
1	-5	0.644	0.065	0.640	0.040	0.004	0.097
1	5	0.594	0.041	0.591	0.094	0.003	0.093
1	10	0.550	0.035	0.500	0.042	0.050	0.041
2	-10	0.624	0.062	0.560	0.076	0.064	0.060
2	-5	0.593	0.031	0.564	0.064	0.029	0.056
2	5	0.561	0.051	0.528	0.036	0.033	0.041
2	10	0.597	0.092	0.572	0.089	0.024	0.059
3	-10	0.842	0.077	0.675	0.122	0.166	0.137
3	-5	0.807	0.070	0.617	0.102	0.190	0.118
3	5	0.828	0.059	0.706	0.098	0.122	0.084
3	10	0.830	0.100	0.652	0.084	0.178	0.097
4	-10	0.671	0.051	0.507	0.058	0.164	0.073
4	-5	0.647	0.042	0.529	0.079	0.118	0.076
4	5	0.634	0.046	0.601	0.092	0.033	0.073
4	10	0.587	0.060	0.533	0.067	0.054	0.036
5	-10	0.689	0.081	0.535	0.066	0.154	0.067
5	-5	0.629	0.062	0.538	0.093	0.092	0.079
5	5	0.622	0.072	0.514	0.059	0.108	0.089
5	10	0.623	0.068	0.517	0.074	0.106	0.071
6	-10	0.567	0.043	0.460	0.065	0.107	0.076
6	-5	0.554	0.098	0.448	0.066	0.106	0.099
6	5	0.559	0.090	0.437	0.105	0.121	0.086
6	10	0.506	0.043	0.389	0.062	0.118	0.064
7	-10	0.686	0.058	0.624	0.118	0.061	0.125
7	-5	0.663	0.071	0.549	0.119	0.114	0.123
7	5	0.660	0.078	0.585	0.096	0.075	0.119
7	10	0.623	0.060	0.551	0.068	0.071	0.055
8	-10	0.769	0.070	0.693	0.075	0.076	0.089
8	-5	0.712	0.059	0.662	0.090	0.049	0.111
8	5	0.690	0.094	0.638	0.090	0.052	0.088
8	10	0.669	0.055	0.660	0.119	0.009	0.123
9	-10	0.660	0.043	0.592	0.078	0.068	0.090
9	-5	0.633	0.056	0.545	0.106	0.088	0.085
9	5	0.585	0.059	0.605	0.089	-0.020	0.107
9	10	0.538	0.084	0.545	0.104	-0.006	0.098
10	-10	0.468	0.026	0.453	0.054	0.015	0.059
10	-5	0.456	0.039	0.404	0.062	0.052	0.039
10	5	0.447	0.037	0.459	0.048	-0.012	0.034
10	10	0.438	0.044	0.434	0.046	0.004	0.037
11	-10	0.674	0.043	0.591	0.156	0.083	0.153
11	-5	0.663	0.046	0.577	0.053	0.087	0.052
11	5	0.660	0.075	0.582	0.063	0.078	0.061
11	10	0.675	0.106	0.618	0.101	0.058	0.045

12	-10	0.470	0.044	0.352	0.044	0.118	0.051
12	-5	0.493	0.027	0.420	0.061	0.073	0.037
12	5	0.455	0.035	0.395	0.052	0.060	0.034
12	10	0.451	0.028	0.391	0.051	0.060	0.055
13	-10	0.553	0.046	0.460	0.104	0.093	0.058
13	-5	0.537	0.031	0.497	0.074	0.040	0.050
13	5	0.540	0.054	0.482	0.087	0.058	0.109
13	10	0.523	0.065	0.435	0.033	0.088	0.064
14	5	0.511	0.028	0.508	0.041	0.004	0.035
14	10	0.501	0.045	0.500	0.048	0.001	0.053

		Peak velocity (m/s)					
		Optotrak		LMC		Difference	
Participant	Location	Mean	Std Dev	Mean	Std Dev	Mean	Std Dev
1	-10	1.049	0.040	0.932	0.043	0.117	0.049
1	-5	1.046	0.100	1.098	0.201	-0.052	0.114
1	5	1.163	0.061	1.107	0.071	0.056	0.046
1	10	1.258	0.126	1.168	0.113	0.090	0.026
2	-10	0.969	0.046	1.090	0.176	-0.121	0.176
2	-5	0.980	0.066	1.099	0.197	-0.119	0.220
2	5	1.021	0.084	1.172	0.260	-0.151	0.216
2	10	1.048	0.078	1.035	0.197	0.013	0.151
3	-10	0.756	0.066	0.833	0.085	-0.077	0.068
3	-5	0.849	0.096	0.901	0.148	-0.052	0.110
3	5	0.848	0.086	0.862	0.113	-0.014	0.081
3	10	0.925	0.075	0.939	0.120	-0.014	0.106
4	-10	1.051	0.063	1.028	0.085	0.023	0.051
4	-5	1.157	0.065	1.140	0.097	0.017	0.061
4	5	1.193	0.052	1.156	0.062	0.037	0.038
4	10	1.240	0.040	1.149	0.103	0.091	0.082
5	-10	0.968	0.138	1.100	0.127	-0.132	0.082
5	-5	0.915	0.144	1.161	0.106	-0.246	0.142
5	5	0.968	0.132	1.169	0.143	-0.201	0.146
5	10	0.922	0.078	1.152	0.121	-0.230	0.135
6	-10	1.091	0.105	1.299	0.162	-0.208	0.100
6	-5	1.190	0.105	1.403	0.216	-0.213	0.158
6	5	1.195	0.150	1.405	0.252	-0.211	0.194
6	10	1.294	0.141	1.463	0.225	-0.169	0.197
7	-10	0.870	0.070	0.930	0.089	-0.060	0.049
7	-5	0.889	0.083	0.928	0.089	-0.039	0.046
7	5	0.951	0.109	0.969	0.118	-0.017	0.077
7	10	0.987	0.048	1.022	0.107	-0.036	0.079
8	-10	0.851	0.058	1.069	0.091	-0.218	0.073
8	-5	0.879	0.056	1.086	0.085	-0.207	0.062
8	5	0.889	0.094	1.131	0.112	-0.242	0.088
8	10	0.926	0.089	1.154	0.116	-0.228	0.050
9	-10	1.118	0.091	1.076	0.115	0.041	0.095
9	-5	1.117	0.125	1.113	0.166	0.004	0.091
9	5	1.208	0.144	1.115	0.195	0.093	0.117
9	10	1.264	0.104	1.243	0.126	0.021	0.049
10	-10	1.239	0.061	1.400	0.060	-0.161	0.081
10	-5	1.251	0.126	1.435	0.220	-0.184	0.117
10	5	1.288	0.081	1.439	0.185	-0.151	0.146
10	10	1.289	0.095	1.505	0.119	-0.217	0.052
11	-10	0.927	0.132	0.954	0.129	-0.027	0.058
11	-5	0.965	0.064	0.986	0.091	-0.021	0.140
11	5	0.927	0.087	0.992	0.185	-0.065	0.162
11	10	0.901	0.158	0.960	0.191	-0.059	0.071

12	-10	1.348	0.104	1.687	0.191	-0.339	0.154
12	-5	1.295	0.108	1.517	0.216	-0.223	0.186
12	5	1.390	0.135	1.641	0.128	-0.251	0.134
12	10	1.413	0.086	1.562	0.210	-0.149	0.207
13	-10	1.128	0.164	1.302	0.251	-0.174	0.184
13	-5	1.135	0.063	1.262	0.154	-0.127	0.168
13	5	1.229	0.080	1.364	0.155	-0.135	0.167
13	10	1.230	0.111	1.416	0.196	-0.186	0.208
14	5	1.153	0.069	1.253	0.114	-0.101	0.128
14	10	1.303	0.070	1.354	0.131	-0.051	0.103

		Duration of acceleration (s)					
		Optotrak		LMC		Difference	
Participant	Location	Mean	Std Dev	Mean	Std Dev	Mean	Std Dev
1	-10	0.288	0.028	0.243	0.020	0.045	0.028
1	-5	0.260	0.035	0.252	0.018	0.008	0.030
1	5	0.249	0.023	0.217	0.024	0.031	0.023
1	10	0.233	0.010	0.197	0.015	0.037	0.020
2	-10	0.284	0.039	0.190	0.052	0.094	0.054
2	-5	0.279	0.024	0.170	0.041	0.109	0.040
2	5	0.244	0.040	0.166	0.029	0.078	0.056
2	10	0.252	0.043	0.202	0.053	0.050	0.051
3	-10	0.305	0.082	0.278	0.089	0.026	0.079
3	-5	0.290	0.077	0.241	0.044	0.049	0.081
3	5	0.309	0.080	0.266	0.055	0.044	0.065
3	10	0.318	0.100	0.250	0.072	0.068	0.060
4	-10	0.318	0.032	0.284	0.044	0.035	0.054
4	-5	0.316	0.032	0.298	0.041	0.018	0.046
4	5	0.294	0.031	0.279	0.044	0.016	0.028
4	10	0.274	0.032	0.269	0.041	0.006	0.032
5	-10	0.286	0.024	0.215	0.057	0.071	0.046
5	-5	0.298	0.027	0.206	0.045	0.092	0.038
5	5	0.280	0.057	0.200	0.048	0.080	0.078
5	10	0.257	0.023	0.189	0.031	0.068	0.049
6	-10	0.248	0.045	0.213	0.043	0.035	0.064
6	-5	0.246	0.042	0.233	0.047	0.014	0.050
6	5	0.234	0.050	0.193	0.036	0.041	0.040
6	10	0.221	0.032	0.180	0.023	0.041	0.037
7	-10	0.327	0.052	0.291	0.077	0.036	0.089
7	-5	0.317	0.049	0.249	0.117	0.068	0.099
7	5	0.296	0.065	0.259	0.094	0.038	0.108
7	10	0.257	0.038	0.219	0.051	0.039	0.047
8	-10	0.383	0.058	0.299	0.055	0.084	0.055
8	-5	0.348	0.029	0.291	0.068	0.058	0.068
8	5	0.339	0.048	0.284	0.050	0.054	0.060
8	10	0.328	0.035	0.311	0.099	0.017	0.097
9	-10	0.205	0.015	0.202	0.026	0.003	0.027
9	-5	0.218	0.074	0.208	0.072	0.010	0.037
9	5	0.218	0.060	0.203	0.062	0.015	0.057
9	10	0.194	0.036	0.183	0.035	0.011	0.021
10	-10	0.245	0.015	0.240	0.023	0.005	0.027
10	-5	0.246	0.030	0.216	0.060	0.030	0.041
10	5	0.218	0.016	0.221	0.032	-0.004	0.028
10	10	0.212	0.019	0.194	0.021	0.018	0.027
11	-10	0.320	0.046	0.289	0.070	0.031	0.054
11	-5	0.307	0.047	0.263	0.092	0.043	0.074
11	5	0.313	0.027	0.221	0.074	0.092	0.077
11	10	0.278	0.043	0.270	0.048	0.008	0.018

12	-10	0.234	0.019	0.166	0.030	0.068	0.039
12	-5	0.247	0.048	0.197	0.053	0.050	0.017
12	5	0.238	0.046	0.208	0.037	0.030	0.035
12	10	0.223	0.014	0.189	0.020	0.034	0.019
13	-10	0.280	0.035	0.240	0.040	0.040	0.020
13	-5	0.257	0.048	0.226	0.038	0.031	0.055
13	5	0.258	0.059	0.246	0.038	0.012	0.065
13	10	0.240	0.052	0.200	0.039	0.040	0.047
14	5	0.225	0.020	0.203	0.022	0.023	0.023
14	10	0.229	0.021	0.221	0.031	0.008	0.022

		Duration of deceleration (s)					
		Optotrak		LMC		Difference	
Participant	Location	Mean	Std Dev	Mean	Std Dev	Mean	Std Dev
1	-10	0.348	0.080	0.263	0.045	0.085	0.080
1	-5	0.384	0.043	0.388	0.054	-0.004	0.079
1	5	0.346	0.032	0.374	0.082	-0.029	0.086
1	10	0.317	0.037	0.303	0.034	0.013	0.039
2	-10	0.340	0.076	0.370	0.066	-0.030	0.063
2	-5	0.314	0.036	0.394	0.057	-0.080	0.054
2	5	0.318	0.057	0.362	0.047	-0.045	0.044
2	10	0.344	0.077	0.370	0.067	-0.026	0.055
3	-10	0.537	0.099	0.397	0.084	0.140	0.121
3	-5	0.517	0.068	0.376	0.091	0.141	0.124
3	5	0.519	0.054	0.440	0.090	0.079	0.086
3	10	0.512	0.043	0.402	0.100	0.110	0.100
4	-10	0.353	0.049	0.224	0.061	0.129	0.064
4	-5	0.331	0.023	0.231	0.064	0.100	0.073
4	5	0.340	0.038	0.323	0.083	0.017	0.073
4	10	0.313	0.034	0.264	0.036	0.049	0.032
5	-10	0.403	0.082	0.320	0.045	0.083	0.090
5	-5	0.332	0.054	0.332	0.070	0.000	0.076
5	5	0.342	0.072	0.314	0.040	0.028	0.070
5	10	0.366	0.075	0.328	0.071	0.038	0.057
6	-10	0.319	0.052	0.247	0.075	0.072	0.090
6	-5	0.308	0.074	0.215	0.051	0.093	0.085
6	5	0.324	0.056	0.244	0.096	0.080	0.090
6	10	0.285	0.049	0.209	0.056	0.076	0.067
7	-10	0.359	0.046	0.333	0.068	0.026	0.068
7	-5	0.346	0.051	0.300	0.064	0.046	0.075
7	5	0.364	0.051	0.326	0.061	0.038	0.052
7	10	0.366	0.049	0.333	0.052	0.033	0.058
8	-10	0.387	0.027	0.395	0.069	-0.008	0.064
8	-5	0.364	0.053	0.372	0.070	-0.008	0.101
8	5	0.351	0.059	0.353	0.063	-0.002	0.056
8	10	0.341	0.044	0.349	0.061	-0.008	0.073
9	-10	0.455	0.036	0.390	0.076	0.065	0.084
9	-5	0.415	0.056	0.337	0.104	0.078	0.099
9	5	0.368	0.063	0.403	0.064	-0.035	0.085
9	10	0.345	0.076	0.362	0.099	-0.017	0.098
10	-10	0.223	0.021	0.213	0.040	0.010	0.048
10	-5	0.210	0.024	0.188	0.027	0.022	0.030
10	5	0.229	0.032	0.238	0.041	-0.008	0.035
10	10	0.226	0.045	0.240	0.040	-0.014	0.035
11	-10	0.354	0.046	0.303	0.197	0.051	0.188
11	-5	0.357	0.046	0.313	0.107	0.043	0.096
11	5	0.347	0.067	0.361	0.070	-0.014	0.034
11	10	0.398	0.088	0.348	0.097	0.050	0.047

12	-10	0.236	0.037	0.186	0.028	0.050	0.037
12	-5	0.247	0.035	0.223	0.051	0.023	0.043
12	5	0.218	0.027	0.188	0.028	0.030	0.037
12	10	0.229	0.036	0.203	0.042	0.026	0.044
13	-10	0.273	0.042	0.220	0.072	0.053	0.042
13	-5	0.280	0.038	0.271	0.083	0.009	0.096
13	5	0.282	0.035	0.236	0.079	0.046	0.077
13	10	0.283	0.031	0.235	0.053	0.048	0.051
14	5	0.286	0.032	0.305	0.039	-0.019	0.035
14	10	0.273	0.046	0.279	0.043	-0.006	0.043

		Endpoint accuracy error (mm)					
		Optotrak		LMC		Difference	
Participant	Location	Mean	Std Dev	Mean	Std Dev	Mean	Std Dev
1	-10	24.554	4.620	54.285	26.325	-29.731	53.453
1	-5	9.436	4.547	38.078	21.542	-28.642	22.696
1	5	10.426	3.569	48.113	21.517	-37.687	20.991
1	10	13.260	2.027	25.383	11.313	-12.123	10.890
2	-10	6.974	3.760	-14.126	5.439	21.101	4.487
2	-5	10.395	6.372	-37.827	8.920	48.222	4.837
2	5	3.828	5.091	-14.196	5.809	18.024	3.858
2	10	2.962	4.329	-7.376	15.795	10.338	14.923
3	-10	5.635	2.591	11.126	15.115	-5.492	15.319
3	-5	2.757	3.565	-2.783	15.709	5.540	15.061
3	5	-2.844	3.769	-30.032	13.622	27.188	14.122
3	10	0.877	5.037	-6.501	24.537	7.378	21.389
4	-10	1.495	3.468	1.374	22.754	0.121	23.048
4	-5	-0.284	3.240	14.650	23.358	-14.934	22.887
4	5	-2.667	6.028	20.864	9.478	-23.531	8.659
4	10	-1.068	5.231	24.274	21.602	-25.342	19.581
5	-10	-12.127	4.085	29.922	21.412	-42.050	21.748
5	-5	-1.894	5.201	10.557	13.451	-12.452	13.960
5	5	-3.750	3.277	27.726	18.589	-31.475	17.792
5	10	-5.226	3.082	29.724	22.936	-34.950	23.364
6	-10	4.477	5.325	-28.720	28.078	33.197	27.398
6	-5	2.893	5.684	-28.843	25.224	31.736	22.653
6	5	2.713	4.913	-26.388	23.385	29.101	23.429
6	10	1.397	4.155	2.220	21.639	-0.823	21.654
7	-10	-2.145	5.102	3.341	8.598	-5.486	7.957
7	-5	-6.835	7.577	-23.359	10.214	16.523	10.285
7	5	-8.668	5.351	-0.038	15.611	-8.630	14.951
7	10	-14.006	4.386	-27.259	16.790	13.253	16.319
8	-10	1.135	3.154	0.350	12.489	0.785	12.625
8	-5	3.315	4.430	-71.841	11.385	75.157	11.582
8	5	10.960	7.865	-10.802	15.893	21.762	17.524
8	10	3.885	4.760	-15.565	9.706	19.450	7.827
9	-10	1.017	3.501	-29.130	9.410	30.147	9.780
9	-5	-1.703	2.549	-4.658	12.317	2.954	12.715
9	5	1.793	3.189	-3.715	21.310	5.508	20.173
9	10	-1.115	2.870	-17.852	10.201	16.737	9.964
10	-10	5.146	2.093	17.008	22.937	-11.862	23.197
10	-5	6.458	3.231	-9.923	14.863	16.381	16.368
10	5	7.715	3.405	-6.271	15.170	13.986	16.439
10	10	6.971	10.642	-9.180	28.160	16.151	20.583
11	-10	11.347	8.464	-51.430	20.771	62.777	22.318
11	-5	21.913	30.873	-22.893	31.163	44.807	9.273
11	5	22.435	8.043	0.615	3.690	21.820	6.615
11	10	14.048	7.201	3.528	15.001	10.520	13.572

12	-10	-1.277	6.830	37.559	17.233	-38.836	14.250
12	-5	-12.995	6.392	-17.137	18.596	4.142	15.258
12	5	-3.328	8.659	13.115	21.789	-16.443	21.970
12	10	-3.656	4.758	-9.100	27.351	5.444	25.724
13	-10	10.753	7.527	-7.713	23.885	18.467	17.016
13	-5	3.824	8.541	-0.481	40.620	4.306	39.534
13	5	-0.588	5.416	-8.340	15.260	7.752	14.012
13	10	8.138	8.941	-17.378	39.656	25.515	40.501
14	5	-7.064	5.285	12.188	17.815	-19.252	20.057
14	10	-5.608	4.036	-10.056	10.077	4.448	10.693
