# Supplementary material for: Evaluation of the Leap Motion Controller during the performance of visually-guided upper limb movements
Source: PLoS One. 2018 Mar 12;13(3):e0193639. doi: 10.1371/journal.pone.0193639 (PMC5846796; doi:10.1371/journal.pone.0193639)
Supplement: S2 Table — Individual subject data averaged across the experimental conditions. (PDF) [file pone.0193639.s002.pdf]

			Movement time (s)					
			Optotrak		LMC		Difference	
Participant	Location	Target ID	Mean	Std Dev	Mean	Std Dev	Mean	Std Dev
1	-10	1	0.463	0.085	0.410	0.141	0.057	0.059
1	-10	2	0.507	0.079	0.393	0.089	0.120	0.052
1	-10	3	0.491	0.048	0.397	0.063	0.095	0.057
1	-10	4	0.498	0.047	0.368	0.046	0.128	0.059
1	10	1	0.432	0.027	0.407	0.076	0.040	0.053
1	10	2	0.491	0.045	0.448	0.076	0.048	0.081
1	10	3	0.466	0.056	0.374	0.069	0.074	0.025
1	10	4	0.498	0.063	0.449	0.072	0.060	0.062
2	-10	1	0.642	0.023	0.590	0.118	0.070	0.115
2	-10	2	0.686	0.069	0.625	0.214	0.070	0.159
2	-10	3	0.676	0.036	0.613	0.101	0.047	0.061
2	-10	4	0.700	0.079	0.544	0.064	0.132	0.093
2	10	1	0.630	0.046	0.508	0.109	0.140	0.101
2	10	2	0.644	0.030	0.580	0.136	0.060	0.115
2	10	3	0.650	0.055	0.523	0.071	0.140	0.095
2	10	4	0.646	0.038	0.480	0.071	0.176	0.091
3	-10	1	0.523	0.100	0.450	0.048	0.100	0.166
3	-10	2	0.506	0.049	0.467	0.083	0.043	0.045
3	-10	3	0.513	0.064	0.454	0.091	0.054	0.066
3	-10	4	0.523	0.066	0.463	0.061	0.073	0.047
3	10	1	0.476	0.046	0.463	0.120	0.020	0.135
3	10	2	0.482	0.043	0.455	0.089	0.035	0.094
3	10	3	0.472	0.044	0.500	0.113	-0.027	0.132
3	10	4	0.491	0.043	0.420	0.086	0.068	0.081
4	-10	1	0.600	0.074	0.553	0.048	0.050	0.078
4	-10	2	0.594	0.048	0.532	0.088	0.060	0.084
4	-10	3	0.602	0.068	0.503	0.064	0.083	0.080
4	-10	4	0.584	0.045	0.620	0.053	-0.027	0.050
4	10	1	0.553	0.044	0.515	0.077	0.050	0.026
4	10	2	0.596	0.080	0.528	0.054	0.052	0.050
4	10	3	0.558	0.076	0.528	0.046	0.040	0.097
4	10	4	0.582	0.048	0.490	0.063	0.087	0.085
5	-10	1	0.688	0.054	0.553	0.105	0.113	0.074
5	-10	2	0.726	0.053	0.607	0.096	0.118	0.076
5	-10	3	0.706	0.061	0.576	0.038	0.108	0.069
5	-10	4	0.683	0.053	0.624	0.099	0.064	0.097
5	10	1	0.677	0.058	0.576	0.100	0.104	0.082
5	10	2	0.700	0.095	0.650	0.156	0.068	0.155
5	10	3	0.672	0.099	0.580	0.080	0.097	0.130
5	10	4	0.660	0.051	0.576	0.089	0.100	0.084
6	-10	1	0.722	0.075	0.611	0.069	0.109	0.102
6	-10	2	0.650	0.088	0.628	0.096	0.033	0.045
6	-10	3	0.660	0.050	0.622	0.095	0.038	0.108
6	-10	4	0.672	0.054	0.650	0.059	0.025	0.042

6	10	1	0.616	0.039	0.589	0.095	0.020	0.086
6	10	2	0.602	0.073	0.589	0.050	0.013	0.073
6	10	3	0.589	0.054	0.556	0.082	0.033	0.112
6	10	4	0.596	0.067	0.575	0.091	0.038	0.079
7	-10	1	0.533	0.071	0.473	0.060	0.073	0.093
7	-10	2	0.492	0.032	0.410	0.048	0.083	0.065
7	-10	3	0.584	0.068	0.460	0.110	0.117	0.092
7	-10	4	0.568	0.089	0.407	0.085	0.127	0.090
7	10	1	0.476	0.052	0.367	0.095	0.113	0.068
7	10	2	0.488	0.048	0.428	0.083	0.068	0.091
7	10	3	0.527	0.042	0.440	0.099	0.088	0.106
7	10	4	0.532	0.080	0.442	0.096	0.087	0.111
8	-10	1	0.566	0.069	0.540	0.097	0.020	0.057
8	-10	2	0.588	0.082	0.529	0.067	0.069	0.062
8	-10	3	0.556	0.086	0.535	0.085	0.033	0.057
8	-10	4	0.548	0.068	0.497	0.069	0.063	0.027
8	10	1	0.626	0.102	0.540	0.103	0.100	0.116
8	10	2	0.650	0.038	0.564	0.139	0.088	0.169
8	10	3	0.733	0.133	0.503	0.082	0.209	0.088
8	10	4	0.663	0.081	0.570	0.081	0.097	0.090
9	-10	1	0.478	0.057	0.411	0.091	0.069	0.084
9	-10	2	0.495	0.087	0.415	0.060	0.080	0.049
9	-10	3	0.470	0.056	0.427	0.070	0.040	0.087
9	-10	4	0.470	0.043	0.404	0.116	0.064	0.107
9	10	1	0.480	0.144	0.400	0.095	0.040	0.082
9	10	2	0.447	0.033	0.384	0.074	0.072	0.052
9	10	3	0.454	0.032	0.424	0.048	0.032	0.030
9	10	4	0.446	0.046	0.403	0.060	0.043	0.051
10	-10	1	0.584	0.055	0.583	0.086	-0.005	0.118
10	-10	2	0.584	0.026	0.570	0.060	0.018	0.072
10	-10	3	0.625	0.080	0.670	0.101	-0.045	0.075
10	-10	4	0.631	0.066	0.648	0.064	-0.020	0.078
10	10	1	0.530	0.024	0.529	0.036	0.003	0.027
10	10	2	0.550	0.041	0.537	0.070	0.013	0.056
10	10	3	0.551	0.027	0.545	0.062	0.008	0.059
10	10	4	0.614	0.064	0.616	0.069	0.004	0.052
11	-10	1	0.490	0.058	0.509	0.058	-0.009	0.016
11	-10	2	0.531	0.076	0.530	0.045	-0.003	0.039
11	-10	3	0.569	0.070	0.554	0.060	0.003	0.027
11	-10	4	0.548	0.043	0.557	0.037	0.003	0.020
11	10	1	0.464	0.026	0.471	0.034	-0.006	0.022
11	10	2	0.488	0.045	0.494	0.073	-0.011	0.045
11	10	3	0.492	0.046	0.516	0.062	-0.012	0.011
11	10	4	0.526	0.070	0.531	0.090	-0.006	0.025
12	-10	1	0.478	0.041	0.550	0.102	-0.057	0.087
12	-10	2	0.489	0.049	0.500	0.080	-0.004	0.067
12	-10	3	0.520	0.094	0.517	0.069	-0.013	0.035

12	-10	4	0.576	0.077	0.540	0.072	0.073	0.083
12	10	1	0.438	0.044	0.460	0.032	-0.012	0.027
12	10	2	0.496	0.072	0.465	0.076	-0.005	0.019
12	10	3	0.474	0.039	0.496	0.065	-0.022	0.049
12	10	4	0.500	0.096	0.520	0.100	-0.033	0.050
13	-10	1	0.442	0.032	0.444	0.036	-0.004	0.048
13	-10	2	0.458	0.048	0.430	0.055	0.028	0.041
13	-10	3	0.468	0.093	0.463	0.084	0.029	0.045
13	-10	4	0.520	0.102	0.424	0.056	0.108	0.124
13	10	1	0.414	0.041	0.453	0.078	-0.060	0.100
13	10	2	0.420	0.052	0.430	0.060	0.000	0.025
13	10	3	0.471	0.095	0.453	0.062	0.037	0.151
13	10	4	0.523	0.123	0.483	0.099	0.060	0.102
14	-10	1	0.571	0.065	0.528	0.108	0.068	0.090
14	-10	2	0.616	0.101	0.567	0.145	0.073	0.117
14	-10	3	0.591	0.070	0.528	0.157	0.060	0.205
14	-10	4	0.600	0.056	0.517	0.078	0.067	0.097
14	10	1	0.580	0.059	0.508	0.050	0.080	0.076
14	10	2	0.570	0.059	0.500	0.049	0.043	0.023
14	10	3	0.604	0.052	0.548	0.086	0.024	0.101
14	10	4	0.630	0.081	0.555	0.111	0.080	0.063
15	-10	1	0.466	0.040	0.475	0.034	-0.010	0.020
15	-10	2	0.465	0.032	0.475	0.030	-0.005	0.019
15	-10	3	0.450	0.072	0.454	0.080	0.000	0.016
15	-10	4	0.458	0.041	0.470	0.054	-0.009	0.024
15	10	1	0.442	0.038	0.451	0.038	-0.011	0.020
15	10	2	0.438	0.046	0.464	0.033	-0.012	0.023
15	10	3	0.440	0.055	0.483	0.056	-0.047	0.053
15	10	4	0.436	0.054	0.447	0.063	-0.022	0.049

			Peak velocity (m/s)					
			Optotrak		LMC		Difference	
**Participant**	**Location**	**Target ID**	**Mean**	**Std Dev**	**Mean**	**Std Dev**	**Mean**	**Std Dev**
1	-10	1	1.508	0.192	1.564	0.302	-0.055	0.154
1	-10	2	1.404	0.153	1.482	0.175	-0.091	0.060
1	-10	3	1.410	0.121	1.430	0.141	-0.021	0.069
1	-10	4	1.448	0.134	1.453	0.106	0.004	0.089
1	10	1	1.580	0.147	1.697	0.105	-0.170	0.226
1	10	2	1.411	0.103	1.504	0.091	-0.078	0.141
1	10	3	1.534	0.179	1.704	0.149	-0.109	0.133
1	10	4	1.391	0.197	1.466	0.208	-0.115	0.126
2	-10	1	0.864	0.055	0.823	0.052	0.072	0.017
2	-10	2	0.823	0.071	0.750	0.104	0.085	0.057
2	-10	3	0.833	0.053	0.753	0.086	0.092	0.061
2	-10	4	0.826	0.049	0.799	0.134	0.056	0.157
2	10	1	0.911	0.080	0.838	0.105	0.084	0.031
2	10	2	0.914	0.075	0.808	0.115	0.103	0.037
2	10	3	0.873	0.096	0.787	0.142	0.057	0.068
2	10	4	0.924	0.065	0.862	0.123	0.066	0.079
3	-10	1	1.163	0.096	1.227	0.042	-0.089	0.180
3	-10	2	1.082	0.102	1.173	0.103	-0.108	0.092
3	-10	3	1.109	0.195	1.177	0.084	-0.067	0.158
3	-10	4	1.086	0.185	1.184	0.132	-0.158	0.122
3	10	1	1.224	0.073	1.148	0.168	0.054	0.175
3	10	2	1.156	0.129	1.231	0.187	-0.080	0.173
3	10	3	1.228	0.161	1.222	0.202	-0.001	0.076
3	10	4	1.163	0.091	1.277	0.172	-0.089	0.067
4	-10	1	0.868	0.044	0.808	0.080	0.078	0.076
4	-10	2	0.855	0.086	0.832	0.072	0.051	0.124
4	-10	3	0.871	0.125	0.799	0.150	0.030	0.095
4	-10	4	0.921	0.101	0.826	0.091	0.068	0.038
4	10	1	0.930	0.050	0.822	0.063	0.107	0.076
4	10	2	0.906	0.072	0.797	0.051	0.100	0.023
4	10	3	1.002	0.128	0.874	0.181	0.147	0.035
4	10	4	0.894	0.073	0.795	0.083	0.118	0.057
5	-10	1	0.857	0.068	0.757	0.054	0.124	0.056
5	-10	2	0.834	0.053	0.757	0.115	0.075	0.110
5	-10	3	0.804	0.017	0.657	0.055	0.147	0.066
5	-10	4	0.839	0.042	0.733	0.039	0.112	0.055
5	10	1	0.950	0.077	0.875	0.100	0.098	0.095
5	10	2	0.903	0.088	0.744	0.084	0.145	0.076
5	10	3	0.968	0.096	0.827	0.133	0.158	0.111
5	10	4	0.958	0.063	0.778	0.088	0.166	0.070
6	-10	1	0.766	0.075	0.834	0.089	-0.060	0.090
6	-10	2	0.826	0.125	0.893	0.168	-0.075	0.078
6	-10	3	0.754	0.082	0.822	0.076	-0.068	0.043
6	-10	4	0.753	0.059	0.808	0.068	-0.054	0.048

6	10	1	0.910	0.129	0.963	0.111	-0.033	0.067
6	10	2	0.889	0.118	0.967	0.105	-0.074	0.078
6	10	3	0.927	0.129	0.946	0.092	-0.019	0.082
6	10	4	0.912	0.092	0.956	0.066	-0.066	0.055
7	-10	1	1.103	0.063	0.982	0.120	0.116	0.098
7	-10	2	1.145	0.083	0.945	0.140	0.213	0.092
7	-10	3	1.083	0.096	0.945	0.097	0.154	0.077
7	-10	4	1.155	0.075	1.042	0.097	0.138	0.102
7	10	1	1.181	0.127	1.067	0.121	0.109	0.147
7	10	2	1.284	0.088	1.177	0.196	0.096	0.143
7	10	3	1.213	0.066	1.032	0.143	0.184	0.131
7	10	4	1.241	0.082	1.175	0.199	0.064	0.214
8	-10	1	1.109	0.104	1.102	0.122	0.044	0.094
8	-10	2	1.053	0.103	0.978	0.128	0.077	0.043
8	-10	3	1.053	0.168	1.104	0.206	-0.036	0.088
8	-10	4	1.108	0.147	1.125	0.209	-0.033	0.137
8	10	1	1.024	0.139	0.998	0.157	0.007	0.062
8	10	2	0.954	0.087	1.023	0.113	-0.056	0.127
8	10	3	0.977	0.102	0.983	0.151	-0.007	0.093
8	10	4	0.933	0.090	0.957	0.108	-0.029	0.120
9	-10	1	1.034	0.125	1.046	0.194	-0.015	0.154
9	-10	2	0.922	0.088	0.855	0.199	0.067	0.150
9	-10	3	0.928	0.097	0.841	0.089	0.100	0.066
9	-10	4	0.979	0.095	1.022	0.181	-0.028	0.174
9	10	1	1.103	0.089	1.068	0.074	0.019	0.159
9	10	2	1.073	0.086	1.122	0.136	-0.071	0.118
9	10	3	1.021	0.098	1.104	0.105	-0.105	0.046
9	10	4	1.047	0.065	1.032	0.107	0.004	0.116
10	-10	1	1.209	0.071	1.194	0.080	0.022	0.065
10	-10	2	1.190	0.080	1.115	0.124	0.086	0.067
10	-10	3	1.160	0.108	1.118	0.097	0.042	0.034
10	-10	4	1.050	0.078	1.002	0.110	0.044	0.066
10	10	1	1.361	0.076	1.284	0.052	0.073	0.057
10	10	2	1.347	0.118	1.188	0.124	0.159	0.153
10	10	3	1.330	0.086	1.274	0.135	0.051	0.057
10	10	4	1.144	0.184	1.073	0.192	0.054	0.068
11	-10	1	1.049	0.115	1.134	0.146	-0.108	0.064
11	-10	2	1.022	0.120	1.135	0.125	-0.113	0.055
11	-10	3	0.955	0.099	1.026	0.073	-0.063	0.032
11	-10	4	0.993	0.094	1.104	0.037	-0.099	0.046
11	10	1	1.095	0.081	1.184	0.126	-0.085	0.061
11	10	2	1.128	0.135	1.156	0.155	-0.007	0.079
11	10	3	1.120	0.188	1.039	0.294	-0.006	0.166
11	10	4	1.093	0.140	1.159	0.198	-0.020	0.064
12	-10	1	1.103	0.162	1.017	0.072	0.002	0.064
12	-10	2	1.160	0.196	1.156	0.169	0.040	0.168
12	-10	3	1.152	0.165	1.102	0.142	0.055	0.091

12	-10	4	1.042	0.138	1.101	0.061	-0.123	0.083
12	10	1	1.324	0.180	1.244	0.198	0.047	0.095
12	10	2	1.173	0.157	1.228	0.173	0.029	0.103
12	10	3	1.213	0.092	1.147	0.181	0.062	0.145
12	10	4	1.217	0.123	1.076	0.129	0.061	0.093
13	-10	1	1.437	0.118	1.323	0.185	0.144	0.143
13	-10	2	1.464	0.096	1.372	0.157	0.065	0.129
13	-10	3	1.335	0.205	1.367	0.215	-0.008	0.236
13	-10	4	1.364	0.210	1.280	0.335	0.103	0.189
13	10	1	1.624	0.129	1.457	0.206	0.203	0.203
13	10	2	1.691	0.157	1.519	0.192	0.164	0.145
13	10	3	1.596	0.104	1.459	0.239	0.139	0.252
13	10	4	1.445	0.312	1.279	0.289	0.062	0.106
14	-10	1	1.079	0.108	1.091	0.140	-0.077	0.078
14	-10	2	1.061	0.089	1.166	0.160	-0.127	0.150
14	-10	3	1.057	0.157	1.119	0.073	-0.014	0.147
14	-10	4	1.028	0.083	1.079	0.126	-0.043	0.067
14	10	1	0.993	0.082	1.013	0.122	-0.061	0.105
14	10	2	1.065	0.071	1.090	0.131	0.005	0.093
14	10	3	0.998	0.089	1.074	0.056	-0.030	0.091
14	10	4	0.977	0.108	0.963	0.119	-0.032	0.025
15	-10	1	1.308	0.122	1.162	0.051	0.090	0.158
15	-10	2	1.314	0.121	1.232	0.098	0.037	0.026
15	-10	3	1.341	0.117	1.261	0.109	0.064	0.070
15	-10	4	1.282	0.092	1.260	0.096	0.016	0.053
15	10	1	1.422	0.090	1.328	0.099	0.106	0.017
15	10	2	1.380	0.097	1.259	0.108	0.084	0.013
15	10	3	1.465	0.138	1.325	0.181	0.132	0.124
15	10	4	1.429	0.107	1.347	0.100	0.097	0.037

			Duration of deceleration (s)					
			Optotrak		LMC		Difference	
Participant	Location	Target ID	Mean	Std Dev	Mean	Std Dev	Mean	Std Dev
1	-10	1	0.257	0.065	0.227	0.135	0.030	0.076
1	-10	2	0.309	0.066	0.213	0.099	0.107	0.065
1	-10	3	0.300	0.049	0.222	0.066	0.078	0.069
1	-10	4	0.306	0.046	0.193	0.045	0.112	0.063
1	10	1	0.244	0.017	0.253	0.070	-0.007	0.050
1	10	2	0.303	0.039	0.256	0.101	0.052	0.109
1	10	3	0.284	0.044	0.211	0.055	0.063	0.024
1	10	4	0.295	0.046	0.257	0.064	0.049	0.050
2	-10	1	0.322	0.031	0.290	0.035	0.035	0.060
2	-10	2	0.366	0.075	0.375	0.050	-0.010	0.048
2	-10	3	0.344	0.047	0.393	0.031	-0.053	0.031
2	-10	4	0.370	0.064	0.320	0.114	0.044	0.110
2	10	1	0.340	0.044	0.268	0.097	0.084	0.078
2	10	2	0.312	0.027	0.267	0.064	0.037	0.075
2	10	3	0.342	0.055	0.306	0.091	0.051	0.089
2	10	4	0.330	0.032	0.248	0.074	0.088	0.094
3	-10	1	0.258	0.055	0.280	0.052	-0.005	0.100
3	-10	2	0.291	0.062	0.280	0.089	0.020	0.057
3	-10	3	0.295	0.062	0.274	0.067	0.023	0.053
3	-10	4	0.293	0.065	0.267	0.068	0.030	0.071
3	10	1	0.249	0.027	0.240	0.052	0.007	0.068
3	10	2	0.266	0.048	0.258	0.048	0.020	0.041
3	10	3	0.264	0.050	0.278	0.070	-0.009	0.079
3	10	4	0.289	0.036	0.236	0.077	0.052	0.067
4	-10	1	0.332	0.050	0.350	0.064	-0.020	0.079
4	-10	2	0.350	0.040	0.344	0.096	-0.004	0.125
4	-10	3	0.370	0.063	0.290	0.069	0.070	0.112
4	-10	4	0.368	0.047	0.427	0.023	-0.027	0.081
4	10	1	0.331	0.063	0.355	0.087	-0.020	0.063
4	10	2	0.364	0.067	0.316	0.050	0.032	0.044
4	10	3	0.330	0.040	0.344	0.052	-0.004	0.048
4	10	4	0.336	0.030	0.273	0.083	0.060	0.107
5	-10	1	0.365	0.040	0.327	0.072	0.023	0.077
5	-10	2	0.404	0.043	0.340	0.032	0.058	0.044
5	-10	3	0.394	0.036	0.380	0.037	-0.004	0.036
5	-10	4	0.369	0.047	0.316	0.054	0.060	0.071
5	10	1	0.349	0.040	0.360	0.062	-0.008	0.041
5	10	2	0.364	0.043	0.388	0.072	-0.015	0.081
5	10	3	0.340	0.063	0.349	0.051	-0.009	0.036
5	10	4	0.348	0.041	0.376	0.120	-0.024	0.121
6	-10	1	0.442	0.043	0.393	0.071	0.051	0.103
6	-10	2	0.408	0.067	0.395	0.082	0.013	0.058
6	-10	3	0.427	0.040	0.373	0.072	0.053	0.088
6	-10	4	0.414	0.078	0.400	0.074	0.025	0.046

6	10	1	0.390	0.036	0.389	0.057	-0.002	0.034
6	10	2	0.374	0.069	0.356	0.057	0.018	0.063
6	10	3	0.384	0.043	0.342	0.034	0.042	0.059
6	10	4	0.374	0.060	0.365	0.069	0.028	0.071
7	-10	1	0.262	0.027	0.270	0.077	-0.003	0.074
7	-10	2	0.254	0.025	0.213	0.030	0.043	0.031
7	-10	3	0.347	0.054	0.286	0.124	0.054	0.090
7	-10	4	0.282	0.044	0.223	0.089	0.060	0.096
7	10	1	0.269	0.038	0.227	0.084	0.047	0.055
7	10	2	0.286	0.038	0.235	0.052	0.055	0.058
7	10	3	0.309	0.020	0.263	0.108	0.045	0.118
7	10	4	0.308	0.054	0.229	0.101	0.071	0.106
8	-10	1	0.294	0.063	0.314	0.071	-0.023	0.041
8	-10	2	0.292	0.051	0.287	0.039	0.004	0.061
8	-10	3	0.290	0.066	0.335	0.075	-0.038	0.063
8	-10	4	0.286	0.056	0.297	0.052	0.000	0.031
8	10	1	0.318	0.065	0.296	0.054	0.012	0.110
8	10	2	0.346	0.046	0.332	0.087	0.020	0.122
8	10	3	0.413	0.122	0.303	0.060	0.080	0.095
8	10	4	0.345	0.060	0.363	0.073	-0.020	0.079
9	-10	1	0.253	0.044	0.217	0.045	0.023	0.060
9	-10	2	0.265	0.094	0.220	0.054	0.045	0.091
9	-10	3	0.280	0.044	0.260	0.106	0.047	0.081
9	-10	4	0.247	0.074	0.176	0.026	0.080	0.080
9	10	1	0.247	0.024	0.225	0.053	0.030	0.050
9	10	2	0.250	0.024	0.180	0.040	0.076	0.052
9	10	3	0.254	0.030	0.224	0.022	0.032	0.023
9	10	4	0.249	0.040	0.243	0.059	0.017	0.043
10	-10	1	0.353	0.049	0.353	0.086	-0.008	0.099
10	-10	2	0.362	0.044	0.348	0.037	0.018	0.058
10	-10	3	0.423	0.058	0.463	0.092	-0.040	0.058
10	-10	4	0.400	0.062	0.415	0.088	-0.023	0.095
10	10	1	0.325	0.021	0.323	0.024	0.003	0.024
10	10	2	0.340	0.025	0.327	0.048	0.013	0.043
10	10	3	0.347	0.024	0.353	0.069	-0.010	0.068
10	10	4	0.394	0.049	0.402	0.060	-0.004	0.034
11	-10	1	0.240	0.076	0.269	0.049	-0.031	0.068
11	-10	2	0.316	0.033	0.303	0.015	0.003	0.023
11	-10	3	0.329	0.060	0.317	0.055	0.003	0.018
11	-10	4	0.326	0.033	0.327	0.030	0.013	0.024
11	10	1	0.251	0.045	0.277	0.024	-0.023	0.037
11	10	2	0.276	0.026	0.271	0.025	0.000	0.026
11	10	3	0.268	0.032	0.292	0.030	-0.048	0.036
11	10	4	0.326	0.044	0.337	0.050	-0.009	0.011
12	-10	1	0.273	0.037	0.287	0.052	-0.003	0.027
12	-10	2	0.291	0.039	0.304	0.079	0.000	0.063
12	-10	3	0.300	0.057	0.307	0.064	-0.007	0.016

12	-10	4	0.360	0.057	0.367	0.058	0.020	0.053
12	10	1	0.236	0.036	0.252	0.039	-0.008	0.041
12	10	2	0.287	0.055	0.255	0.053	0.005	0.019
12	10	3	0.264	0.030	0.289	0.058	-0.022	0.042
12	10	4	0.258	0.044	0.253	0.050	0.027	0.061
13	-10	1	0.246	0.044	0.248	0.033	-0.004	0.026
13	-10	2	0.248	0.049	0.258	0.057	0.005	0.042
13	-10	3	0.284	0.079	0.286	0.085	0.017	0.042
13	-10	4	0.293	0.094	0.256	0.074	0.052	0.135
13	10	1	0.230	0.039	0.223	0.037	-0.007	0.035
13	10	2	0.229	0.050	0.247	0.056	-0.007	0.030
13	10	3	0.284	0.098	0.250	0.037	0.053	0.128
13	10	4	0.325	0.118	0.273	0.074	0.063	0.094
14	-10	1	0.307	0.053	0.296	0.074	0.028	0.070
14	-10	2	0.344	0.083	0.340	0.091	0.010	0.037
14	-10	3	0.344	0.080	0.316	0.144	0.052	0.153
14	-10	4	0.333	0.069	0.310	0.055	0.003	0.071
14	10	1	0.313	0.052	0.300	0.042	0.024	0.048
14	10	2	0.324	0.057	0.273	0.078	0.033	0.048
14	10	3	0.352	0.038	0.376	0.075	-0.044	0.074
14	10	4	0.350	0.093	0.280	0.043	0.050	0.111
15	-10	1	0.303	0.042	0.285	0.034	0.010	0.026
15	-10	2	0.303	0.029	0.290	0.035	0.015	0.025
15	-10	3	0.278	0.071	0.269	0.076	0.014	0.030
15	-10	4	0.288	0.037	0.289	0.043	0.001	0.012
15	10	1	0.284	0.038	0.283	0.042	0.000	0.016
15	10	2	0.273	0.040	0.288	0.041	-0.004	0.017
15	10	3	0.283	0.052	0.300	0.075	-0.023	0.041
15	10	4	0.280	0.052	0.287	0.060	-0.013	0.060
