# Supplementary material for: Evaluation of the Leap Motion Controller during the performance of visually-guided upper limb movements
Source: PLoS One. 2018 Mar 12;13(3):e0193639. doi: 10.1371/journal.pone.0193639 (PMC5846796; doi:10.1371/journal.pone.0193639)
Supplement: S3 Table — Individual subject data averaged across the experimental conditions. (PDF) [file pone.0193639.s003.pdf]

	Movement time - to the bead (s)					
	Optotrak		LMC		Difference	
Participant	Mean	Std Dev	Mean	Std Dev	Mean	Std Dev
1	0.580	0.054	0.553	0.065	0.027	0.048
2	0.624	0.050	0.582	0.054	0.042	0.043
3	0.556	0.049	0.617	0.106	-0.062	0.111
4	0.540	0.040	0.510	0.065	0.030	0.047
5	0.553	0.057	0.533	0.081	0.020	0.072
6	0.493	0.054	0.471	0.067	0.021	0.053
7	0.569	0.059	0.494	0.063	0.075	0.063
8	0.557	0.037	0.524	0.047	0.032	0.043
9	0.540	0.050	0.453	0.066	0.087	0.056
10	0.415	0.034	0.395	0.056	0.020	0.049
11	0.420	0.023	0.441	0.042	-0.020	0.044
12	0.556	0.036	0.552	0.072	0.004	0.065
13	0.390	0.050	0.377	0.055	0.013	0.047
14	0.407	0.027	0.407	0.038	-0.001	0.026
15	0.367	0.044	0.359	0.047	0.008	0.029

	Movement time - to the needle (s)					
	Optotrak		LMC		Difference	
Participant	Mean	Std Dev	Mean	Std Dev	Mean	Std Dev
1	0.512	0.052	0.462	0.062	0.050	0.041
2	0.582	0.080	0.527	0.068	0.055	0.048
3	0.573	0.080	0.531	0.066	0.042	0.067
4	0.558	0.053	0.492	0.058	0.066	0.062
5	0.582	0.071	0.492	0.066	0.091	0.077
6	0.473	0.059	0.437	0.052	0.036	0.068
7	0.473	0.046	0.475	0.061	-0.003	0.053
8	0.524	0.053	0.508	0.077	0.016	0.065
9	0.383	0.062	0.386	0.072	-0.003	0.031
10	0.373	0.026	0.350	0.048	0.022	0.040
11	0.420	0.038	0.455	0.065	-0.034	0.070
12	0.508	0.072	0.454	0.076	0.054	0.025
13	0.376	0.046	0.357	0.040	0.019	0.056
14	0.424	0.047	0.429	0.052	-0.005	0.054
15	0.397	0.040	0.405	0.076	-0.008	0.064

	Peak velocity - to the bead (m/s)					
	**Optotrak**		**LMC**		**Difference**	
**Participant**	**Mean**	**Std Dev**	**Mean**	**Std Dev**	**Mean**	**Std Dev**
1	0.906	0.093	0.717	0.113	0.189	0.085
2	0.810	0.085	0.667	0.086	0.143	0.078
3	0.840	0.074	0.719	0.163	0.120	0.144
4	0.765	0.090	0.651	0.077	0.113	0.081
5	0.758	0.069	0.625	0.069	0.133	0.054
6	0.876	0.081	0.746	0.073	0.130	0.089
7	0.844	0.078	0.776	0.100	0.068	0.076
8	0.773	0.068	0.686	0.131	0.087	0.122
9	0.841	0.056	0.850	0.138	-0.009	0.131
10	0.943	0.064	1.035	0.191	-0.093	0.177
11	0.973	0.061	0.923	0.116	0.050	0.141
12	0.782	0.044	0.805	0.161	-0.024	0.157
13	0.994	0.104	1.006	0.146	-0.012	0.142
14	1.023	0.081	0.981	0.120	0.043	0.089
15	1.179	0.149	1.076	0.147	0.103	0.119

	Peak velocity- to the needle (m/s)					
	**Optotrak**		**LMC**		**Difference**	
**Participant**	**Mean**	**Std Dev**	**Mean**	**Std Dev**	**Mean**	**Std Dev**
1	0.955	0.108	0.824	0.131	0.131	0.110
2	0.823	0.101	0.698	0.114	0.125	0.108
3	0.815	0.101	0.725	0.157	0.091	0.119
4	0.749	0.076	0.664	0.093	0.085	0.098
5	0.721	0.092	0.589	0.066	0.133	0.075
6	0.927	0.068	0.772	0.099	0.155	0.116
7	0.926	0.104	0.884	0.125	0.042	0.113
8	0.823	0.111	0.774	0.173	0.049	0.153
9	1.075	0.138	0.988	0.182	0.087	0.117
10	1.127	0.072	1.080	0.145	0.048	0.131
11	0.997	0.082	0.955	0.158	0.041	0.128
12	0.838	0.088	0.808	0.214	0.030	0.174
13	1.056	0.071	1.036	0.212	0.020	0.206
14	1.023	0.057	0.867	0.072	0.156	0.052
15	1.109	0.072	0.984	0.093	0.126	0.081

	Grasping duration (ms)					
	Optotrak		LMC		Difference	
Participant	Mean	Std Dev	Mean	Std Dev	Mean	Std Dev
1	0.138	0.064	0.195	0.063	-0.057	0.048
2	0.248	0.073	0.316	0.067	-0.068	0.055
3	0.180	0.060	0.193	0.095	-0.012	0.096
4	0.134	0.066	0.201	0.068	-0.067	0.053
5	0.185	0.100	0.268	0.092	-0.082	0.086
6	0.184	0.082	0.226	0.098	-0.042	0.063
7	0.252	0.093	0.283	0.102	-0.031	0.061
8	0.155	0.071	0.193	0.086	-0.038	0.068
9	0.086	0.102	0.150	0.096	-0.064	0.051
10	0.073	0.021	0.105	0.043	-0.032	0.040
11	0.069	0.027	0.082	0.028	-0.013	0.024
12	0.133	0.033	0.161	0.057	-0.028	0.053
13	0.077	0.040	0.106	0.050	-0.029	0.044
14	0.131	0.052	0.159	0.039	-0.029	0.044
15	0.070	0.049	0.095	0.066	-0.025	0.028

	Placement duration (ms)					
	Optotrak		LMC		Difference	
Participant	Mean	Std Dev	Mean	Std Dev	Mean	Std Dev
1	0.483	0.206	0.531	0.208	-0.048	0.074
2	0.867	0.265	0.917	0.283	-0.049	0.090
3	0.683	0.304	0.709	0.305	-0.027	0.135
4	0.400	0.205	0.471	0.224	-0.070	0.073
5	0.443	0.146	0.555	0.145	-0.112	0.053
6	0.501	0.270	0.586	0.289	-0.085	0.116
7	0.436	0.113	0.526	0.118	-0.090	0.065
8	0.447	0.212	0.502	0.196	-0.055	0.083
9	0.508	0.294	0.513	0.271	-0.005	0.073
10	0.313	0.229	0.347	0.221	-0.034	0.050
11	0.332	0.330	0.364	0.313	-0.031	0.079
12	0.309	0.206	0.386	0.214	-0.077	0.062
13	0.246	0.195	0.284	0.192	-0.038	0.071
14	0.263	0.138	0.303	0.148	-0.039	0.066
15	0.355	0.249	0.356	0.244	0.000	0.083
